# Large-scale benchmarking of prokaryotic annotation tools across thousands of species

**DOI:** 10.1186/s13059-026-04262-0

**Published:** 2026-09-04

**Authors:** Mateusz Jundzill, Martin Hölzer, Serghei Mangul, Mike Marquet, Ralf Ehricht, Mara Lohde, Riccardo Spott, Oliwia Makarewicz, Mathias W. Pletz, Christian Brandt

**Affiliations:** 1 Institute for Infectious Diseases and Infection Control, Jena University Hospital, Jena, 07747 Germany; 2 Bioinformatics and Translational Research, Genome Competence Center, Robert Koch Institute, Berlin, 13353 Germany; 3 InfectoGnostics Research Campus, Center for Applied Research, Jena, 07743 Germany; 4 Leibniz Institute of Photonic Technology (IPHT), Jena, 07745 Germany; 5 Leibniz Center for Photonics in Infection Research (LPI), Jena, 07745 Germany; 6 Institute of Physical Chemistry, Friedrich-Schiller-University Jena, Jena, 07743 Germany; 7 Department of Biological and Morphofunctional Sciences, College of Medicine and Biological Sciences, Stefan Cel Mare University of Suceava, Suceava, 720229 Romania; 8 Department of Clinical Pharmacy, Alfred E. Mann School of Pharmacy and Pharmaceutical Sciences, University of Southern California, Los Angeles, CA USA; 9 Department of Computers, Informatics and Microelectronics, Technical University of Moldova, Chisinau, Moldova; 10 Małopolska Centre of Biotechnology, Jagiellonian University, Kraków, Poland; 11 Biological and Life Sciences Division, Mohamed bin Zayed University of Artificial Intelligence, Abu Dhabi, UAE

**Keywords:** Bioinformatics, Genome annotation, Bacteria, Archaea, Benchmark, Computational biology, Metagenome-assembled genome, Functional annotation, Gene function prediction, Gene ontology

## Abstract

**Background:**

Genome annotation is an important step in deriving functional meaning from prokaryotic sequencing data, yet systematic evaluations guiding tool selection are lacking. We present the first large-scale investigation of four prominent open-source annotation tools (Prokka, Bakta, EggNOG-mapper, and PGAP) across 156,033 diverse genomes. This includes *Escherichia coli* strains for baseline performance, thousands of archaea and bacteria genomes, as well as frameshifted and metagenome-assembled genomes.

**Results:**

Bakta excels in annotating high-quality bacterial genomes, while PGAP was better for archaeal genomes and challenging bacterial assemblies, including metagenome-assembled, fragmented, or contaminated samples. For Gene Ontology annotation, PGAP consistently provides broader term coverage, whereas EggNOG-mapper offers more terms per feature.

**Conclusions:**

Our findings highlight tool-specific strengths crucial for selecting optimal solutions based on genome quality, taxonomy, and origin (e.g. MAGs). This study provides an evidence-based guide for users and informs future tool development.

**Supplementary Information:**

The online version contains supplementary material available at https://doi.org/10.1186/s13059-026-04262-0.

## Background

The rapid advancements in genomics and sequencing technologies have continuously improved our understanding of prokaryotic organisms, their genomic landscapes, and features. This progress is crucial as it underpins advancements across various disciplines, like biotechnology, biomedicine, and environmental science. As we move past well-studied genes and organisms, the demand for reliable and precise prokaryotic gene identification by annotation tools becomes increasingly important. Annotation is the cornerstone for gene identification, facilitating expression profiling and downstream applications by identifying and characterizing genomic features.

In current state-of-the-art tools, the annotation process can be divided into two distinct phases. The initial annotation phase involves feature prediction leveraging machine learning algorithms like Hidden Markov model chains to differentiate coding from non-coding regions. Tools such as Prodigal [1], GeneMarkS-2 [2], or Augustus are commonly employed for this purpose, while the latter is mainly used for eukaryotic organisms [3]. After initial annotation, filtering is applied to eliminate dubious or known false positives, although this can lead to the loss of important protein-coding genes below 100 amino acids (aa) [4]. Separately, distinct features, like RNA groups, are predicted using additional tools to improve accuracy.

In the subsequent annotation phase, the focus shifts to assigning a description for each coding sequence (CDS) through either sequence similarity commonly referred to as homology or orthology interference [5, 6]. Although orthology inference is considered more precise, it is computationally more demanding [7]. Various controlled vocabulary databases, including GO, KEGG, CARD, COG, and PFAM facilitate biological functional annotation of genes. Notably, each database has its specialty, such as CARD's focus on antimicrobial resistance genes [8] and PFAM's emphasis on protein domains and evolutionary relationships [9].

Although the annotation process is vital, there is a notable lack of comprehensive comparisons on tool performance and factors influencing it, such as source, taxonomy, or assembly quality. Existing tool comparisons narrow their focus on individual steps [10] or overlook recently developed pipelines [11].

**Fig. 1:**
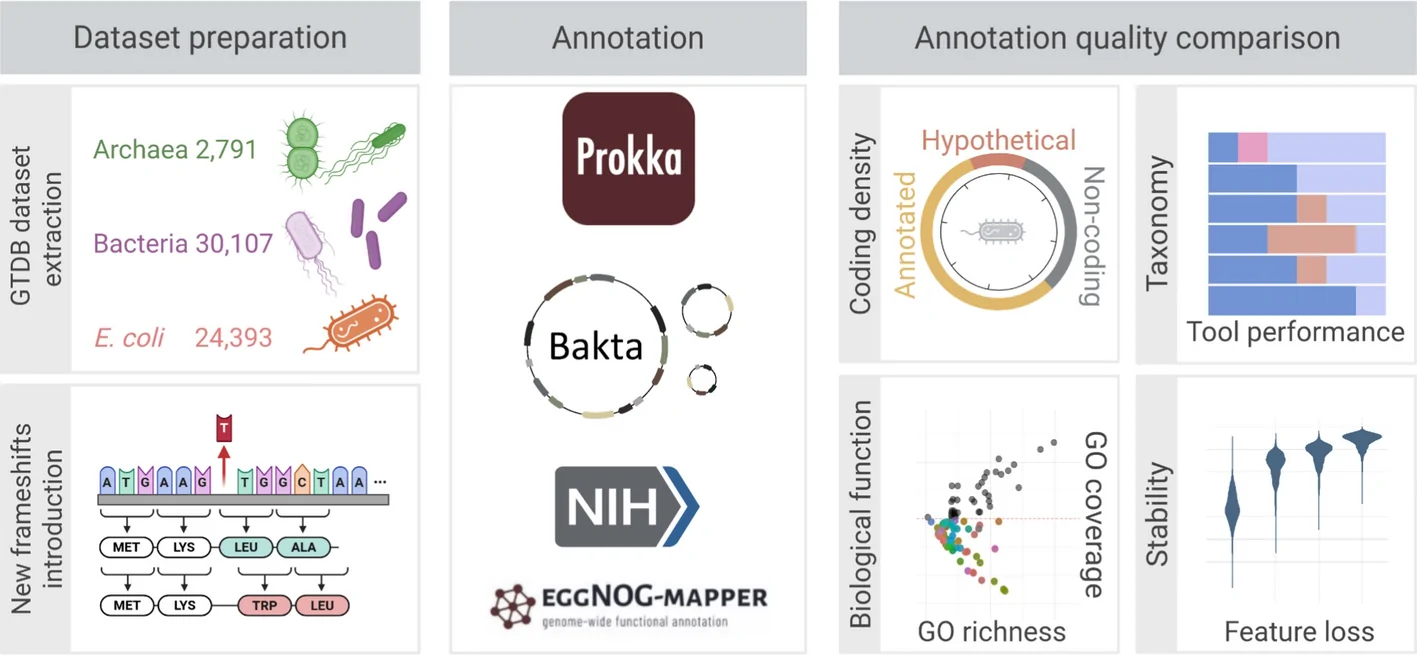
Large-scale evaluation of prokaryotic annotation tools across thousands of species. The Genome Taxonomy Database (GTDB 207.0) was queried for representative Bacteria and Archaea species and all Escherichia coli strains. Additionally, genomes were generated from the representatives to include frameshifts of 0.5%, 1%, and 2%. All genomes were annotated using Prokka, Bakta, EggNOG-mapper, and PGAP with their recommended settings. The annotation quality between tools and genome conditions was compared based on multiple characteristics like coding density, detected feature length, Genome Ontology (GO) richness, and impact of frameshifts. Created in BioRender. Röll, D. (2026) biorender.com/2ufrc7z

Our study systematically evaluates popular, free, and open-source genome annotation tools to provide practical guidelines tailored to taxonomic context, desired annotation features, and error tolerance in Bacteria and Archaea (Fig. 1). We focus on four widely used tools: Prokka [12], Bakta [13], EggNOG-mapper [14], and PGAP [6]. These tools represent a spectrum of annotation strategies, from Prokka's rapid, resource-efficient annotations to Bakta's extensive database coverage. EggNOG-mapper offers robust biological function and orthology-based annotation, including eukaryotes, but focuses on CDS features only. PGAP, used in-house by the National Center for Biotechnology Information (NCBI), stands out for its taxonomy-tailored pipelines, which ensure precise annotations but require prior taxonomic information.

To ensure broad applicability, we curated a diverse dataset using the Genome Taxonomy Database (GTDB), prioritizing its taxonomic accuracy over traditional NCBI datasets [15, 16]. We made sure to incorporate scenarios reflecting real-world challenges, such as metagenome-assembled genomes (MAGs), simulated assembly issues (e.g., frameshifts), and temporal changes in database quality. These comprehensive assessments aim to guide tool selection based on specific needs and also highlight critical limitations to address in future tool developments.

## Results

### Genome data acquisition and annotation tool evaluation

For our large-scale benchmark of prokaryotic annotation tools, we annotated a total of 156,033 genomes to assess each tool's performance under various conditions. 29,761 bacterial and archaeal genomes were retrieved from the GTDB database (release 207.0) and downloaded from the NCBI database (Additional file 1: Fig. S1). Given need for the reference point and the absence of a gold standard for structural and functional annotation in large, taxonomically diverse datasets (Additional file 1: Figs. S2-3), we complement this data with an additional subset of 24,393 *Escherichia coli* strains, which serve as an intentionally conservative, low-taxonomic-variability, and well-researched reference point. Where research-validated expectations exist such as the single-copy nature of tmRNA and the completeness of rRNA operons we used these as guiding benchmarks for annotation quality assessment. To assess annotation database reliability, we evaluated the proportion of features assigned database accessions for UniProtKB (Prokka), UniRef100/50 (Bakta), PFAM (EggNOG-mapper), and RefSeq (PGAP), treating accession-linked descriptions as more reliable.

Furthermore, we created 98,742 genomes to evaluate the impact of frameshifts on annotation quality by introducing pseudo-random genome-wide deletions (0.5%, 1%, and 2%) to a subset of the bacterial genomes, simulating sequencing errors. Additionally, 3,137 MAGs from different families were individually extracted and investigated (Methods: Datasets).

We selected Prokka, Bakta, EggNOG-mapper, and PGAP due to their free and open-source nature and overall popularity (Table 1). For the analysis, we used each tool's recommended settings, except for the adjusted 4th translation table for the phylum Mycoplasmatota (Methods: Tools).

**Table 1:** Overview of the benchmarked tool characteristics. CPU hours were used to adjust for different hardware settings during the benchmarking. The hardware settings used to minimize time and cost for this analysis are available in Methods: Tools; Workflow execution

		Prokka	Bakta	EggNOG-mapper	PGAP
Scope	Coverage	Virus, Archaea, Bacteria, Mitochondria	Bacteria	Virus, Archaea, Bacteria, Eukaryotes	Archaea, Bacteria
	Annotated features (non-RNA)	CDS, Signal leader peptides	CDS, sORF, CRISPR, cis-regulatory regions, oriC/oriV/oriT, assembly gaps	CDS	CDS, CRISPR, Phage-related genes
	RNA features	tRNA, rRNA, ncRNA	tRNA, tmRNA, rRNA, ncRNA	N/A	tRNA, rRNA, ncRNA
	Main functional annotation databases	COG	COG, GO	COG, GO, KEGG pathways, Brite, EC	GO, EC
Prediction	Main prediction tools	Prodigal (CDS), Barnap/RNAmmer (rRNA), Aragorn (tRNA), Infernal (ncRNA)	Prodigal($) (CDS), alignment-free sequence identification (< 29 aa proteins), tRNAscan-SE (tRNA), Aragorn (tmRNA), Infernal (rRNA, ncRNA)	Diamond/MMseqs2/Prodigal (CDS)	ORFfinder/BLAST/GeneMark-S2 + (CDS), Infernal (rRNA; ncRNA), tRNAscan-SE (tRNA)
	Prediction approach	Sequence similarity	Sequence similarity	Orthology	Sequence similarity
Database	Database size (unpacked)[Gb](+)	0.2	59.4	51.0	30.4
	Average tool update time (#)	Last depreciation update on December 2025	165 days	255 days	123 days
Performance	Median CPU time [min](*)	3.2	36.8	15.6	106.6
	Median RAM used [Gb](*)	0.6	8.6	41.7 (*)	4.5
Other	Requires taxonomy	Not required	Not required	Not required	Needs NCBI genus or species name
	Installation(-)	Conda, Docker, Singularity, Brew Integrated database that does not require additional handling	Conda, Docker, Singularity, PyPi Necessary independent database download and update	Conda, PyPi, script Necessary independent database download and tool setup	Docker, script The installation script downloads the database
	Execution	Single command, examples provided in the repository	Single command, examples provided in the repository	Complex single command, examples provided in the repository	Completion of the metadata YAML file is required before command execution
	Additional features	Custom, user-defined feature database	Genome visualization, JSON output, Reduced (light) dataset for faster execution, Custom, user-defined feature database	Option to limit taxonomy scope, High memory mode to improve annotation performance	Auto-adjust taxonomy, Taxonomy checks, Robust annotation correction mechanism, Contamination detection

+ Database size for the versions used for the analysis

^#^Checked from January 2001 to October 2024

^*^Across 9,842 different bacterial genomes; EggNOG-mapper was used with –dbmem flag for speed optimization

^$^Prodigal is used through Pyrodigal Prodigal bindings which produce the same results as Prodigal v2.6.3 + 31b300a

- Only installation methods officially recommended in the GitHub repository are listed

We assessed annotation quality using three primary parameters: coding density (as the proportion of the genome size, if presented as raw length, it is considered as coding space), the count of RNA features (rRNA, tRNA, tmRNA), and biological function annotation via Gene Ontology (GO) terms, among others presented in Additional files 1–2 (see Data analysis; Methods). These parameters are inherently interdependent, as their evaluation directly relies on the capabilities of ORF detection (measured by total coding density). Therefore, they should not be interpreted in isolation from each other.

One of the primary metrics to assess each tool's ability to predict features was measuring the coding density parameters. Total coding density metric evaluates the performance of ORF detection. Total coding density analysis is primarily quantitative, supplemented by a qualitative investigation of specific errors. Readers interested in a qualitative, in-depth analysis of pure ORF detection tools are directed to Dimonaco et al. [10]. While described and undescribed coding density, investigating tools detection and database capabilities. Meanwhile, the non-coding region denotes genome length where no features were detected. Described coding density includes features with known descriptions, while undescribed coding density pertains to hypothetical proteins and features without any descriptions (which may consist of non-coding type features that a particular tool does not detect). This approach minimizes the impact of feature size variations and naming discrepancies, unlike raw feature count. Ideally, tools should display a large, described coding density with minimal undescribed regions and consistent coding density sizes across the same well-studied species, e.g., *E. coli* (Method: Data analysis). The described features typically do not include information on biological processes and should not be confused with the biological function annotations of genes using GO terms.

We compared and quantified each tool's biological function annotation performance by using its assigned GO terms. Two metrics were measured: (1) GO coverage, the proportion of genes annotated with at least one GO term, and (2) GO richness, the number of GO terms per gene. Both metrics were considered, recognizing that, depending on the use case, a single detailed GO term can offer similar functional insights as multiple GO terms. Prokka was excluded from this analysis as it assigns no GO terms; EggNOG-mapper's developer-recommended default settings limit it to curated non-electronic GO terms annotations (Methods: Tools).

### Comprehensive evaluation of genome annotation tools using *Escherichia coli* as a model reveals tool-specific strengths

To assess baseline annotation performance, we evaluated all tools by using 24,393 different *E. coli* genomes, spanning 2,162 known serotypes (Methods: Datasets, Additional file 2: Table S1).

#### Coding density

**Fig. 2:**
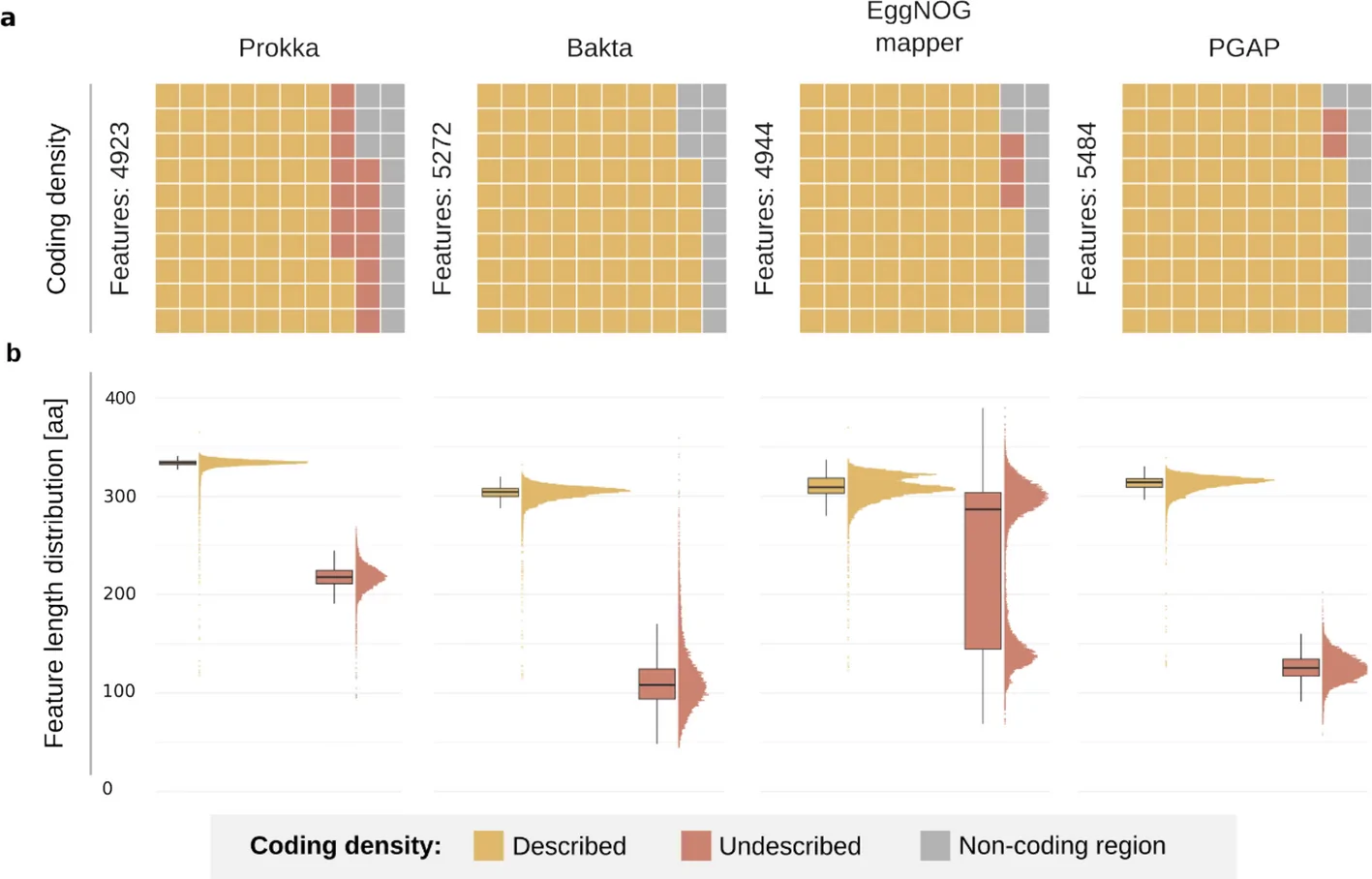
E. coli coding density and gene length distribution. **a** Coding density: The average coding density detected by the tools is categorized into described coding density (yellow), undescribed coding density (red), and non-coding regions (gray). Each square represents 1% of the total genome. The mean feature count is noted on the left. **b** Coding length distribution: The average amino acid length distribution of features for described (yellow) and undescribed (red) coding density is shown

Our initial analysis targeted the total coding density covering described features (yellow area, Fig. 2a), undescribed features (red area, Fig. 2a), and the non-coding density (gray area, Fig. 2a). We also assessed feature lengths for inaccuracies, such as gene merging that leads to unusually long features [17] (Fig. 2b). For reference, the average *E. coli* genome in our dataset spans 5,120,395 base pairs (bp) (standard deviation [SD] = 274,047 bp; Additional file 1: Fig. S4).

When comparing total coding density (sum of described and undescribed space) across the 24,393 *E. coli* genomes, PGAP annotated the largest average total coding space, with a mean of 4,709,183 bp (SD = 301,104 bp), followed by Bakta (mean = 4,497,294 bp, SD = 229,128 bp) and EggNOG-mapper (mean = 4,498,886 bp, SD = 234,135 bp). Prokka exhibited the smallest total coding space (mean = 4,457,521 bp, SD = 223,485 bp).

When considering only the described coding density, Bakta had the largest average, followed by PGAP. Prokka's mean described coding space was substantially lower than that of the other tools (Bakta: mean = 4,485,330 bp, SD = 229,525 bp; PGAP: mean = 4,431,578 bp, SD = 210,208 bp; EggNOG-mapper: mean = 4,347,920 bp, SD = 193,514 bp; Prokka: mean = 3,715,142 bp, SD = 108,640 bp). However, it exhibited the lowest variability, possibly due to its limited feature annotation, as reflected in the smaller described coding density. PGAP had the highest mean proportion of features with database accessions (RefSeq: 92.50%), followed by Bakta (UniRef50: 91.48%, UniRef100: 89.42%), EggNOG-mapper (PFAM: 90.39%), and Prokka (UniProtKB 73.33%). These accession identifiers enable the retrieval of further functional information, independent of the annotation tool database.

When comparing the preferred small undescribed coding density, Bakta demonstrated the smallest across all genomes with the lowest mean and deviation (mean = 11,965 bp, SD = 17,084 bp), followed by PGAP, EggNOG-mapper, and Prokka (PGAP: mean = 109,801 bp, SD = 30,664 bp; EggNOG-mapper: mean = 150,966 bp, SD = 66,604 bp; Prokka: mean = 741,364 bp, SD = 141,688 bp). On a side note, Prokka and EggNOG-mapper generated longer mean undescribed features (Fig. 2b red). EggNOG-mapper may have assigned long noncoding features like assembly gaps as hypothetical proteins (Additional file 1: Fig. S5), which resulted in a bimodal undescribed coding length distribution (Fig. 2b, red EggNOG-mapper). For all comparative analysis between the tools, see Additional file 1: Tables S1-7.

#### RNA features

Next, we evaluated the annotation of RNA features. *E. coli* contains seven operons, each composed of 16S rRNA, 23S rRNA, and 5S rRNA genes, with one additional 5S rRNA, totalling 22 rRNA genes [18, 19]. For simplicity, we considered only the total detected rRNA count, without distinguishing between complete or incomplete operons. EggNOG-mapper was excluded from this analysis as it does not annotate rRNA features.

None of the tools consistently annotated all rRNA operons across the *E. coli* strains. On average, PGAP identified the highest count of rRNA genes (mean = 13.89, SD = 8.36), followed by Prokka (mean = 10.35, SD = 6.10) and Bakta (mean = 9.60, SD = 6.27). The requirement for at least one complete operon was not fulfilled in 638 cases with Prokka, 779 with Bakta, and 451 with PGAP. In genomes with fewer than 10 contigs, all tools consistently annotated a similar high mean of rRNA (Prokka: mean = 21.98, SD = 1.33; Bakta: mean = 21.96, SD = 1.27; PGAP: mean: 21.95, SD = 1.20; Additional file 1: Fig. S6). However, in samples with more fragmented genomes (> 10 contigs), the total annotated rRNA count decreased substantially. PGAP maintained the highest count in these cases (mean = 12.96, SD = 8.33), compared to Prokka and Bakta (Prokka: mean = 8.99, SD = 4.88; Bakta: mean = 8.17, SD = 4.89).

*E. coli* contains 79 tRNA genes [20], and PGAP detected a similar count of these features (mean = 78.74, SD = 12.47). Prokka and Bakta detected, on average, three more features (Prokka: mean = 81.15, SD = 12.54; Bakta: mean = 81.72, SD = 12.59) (Additional file 1: Fig. S6).

Bacteria are generally expected to have a single tmRNA gene per genome (Williams, 2003). All evaluated tools demonstrated a mean tmRNA value of 1 (SD: Bakta: 0.08; PGAP: 0.09; Prokka: 0.07). However, Prokka and Bakta failed to identify at least one tmRNA in 62 cases, while PGAP missed it in 15 genomes. Furthermore, Prokka identified more than one tmRNA in 35 instances, Bakta in 83, and PGAP in 127.

#### Biological functional annotation

To evaluate the performance of functional annotation, we analyzed the GO coverage and GO richness. EggNOG-mapper exhibited the highest GO coverage, with a mean of 66.07% (SD = 4.59%), comparable to Bakta (mean = 63.88%, SD = 5.25%). PGAP showed the lowest average GO coverage of 36.12% (SD = 1.95%). For GO richness, EggNOG-mapper led with a mean of 37.00 terms per gene (SD = 0.48), compared to Bakta (mean = 4.00, SD = 0.02) and PGAP (mean = 2.00, SD = 0.00). It is important to note that EggNOG-mapper identified fewer genes than PGAP (Fig. 2a), which might influence both GO richness and coverage performance (Additional file 2: Table S2). Additionally, we performed a limited quality analysis of GO terms using *Escherichia coli* K-12 as a representative example. To assess the comprehensiveness of the annotations, we measured GO term depth, which varied substantially across different ontologies. However, PGAP and Bakta consistently outperformed other tools in terms of depth across all ontologies (Additional file 1: Table S8), while EggNOG-mapper identified the highest number of unique GO terms (Additional file 1: Fig. S7).

### Investigating annotation performance across 29,761 bacterial and archaeal species: taxonomy-related disparities among tools

Using the performance metrics established for *E. coli*, we evaluated the impact of taxonomic variance on annotation quality across Bacteria and Archaea. This section covers 2,791 archaeal (20 phyla) and 26,970 bacterial (163 phyla) representative species. Characterization of the datasets are presented in Additional file 1: Figs. S8-11. We excluded 3,137 bacterial MAGs and analyzed them separately in the “Assessing Annotation Stability” section, because of their later outlined properties affecting annotation performance. However, due to the low amount of Archaeal non-MAGs (760 non-MAGs to 2,031 MAGs), we decided to include these MAGs to maintain a meaningful Archaea representation (Methods: Datasets). It should be noted that Bakta does not officially support archaeal annotation; therefore, these results should be interpreted with caution and may not be fully comparable to those generated by other tools.

#### Coding density

**Fig. 3:**
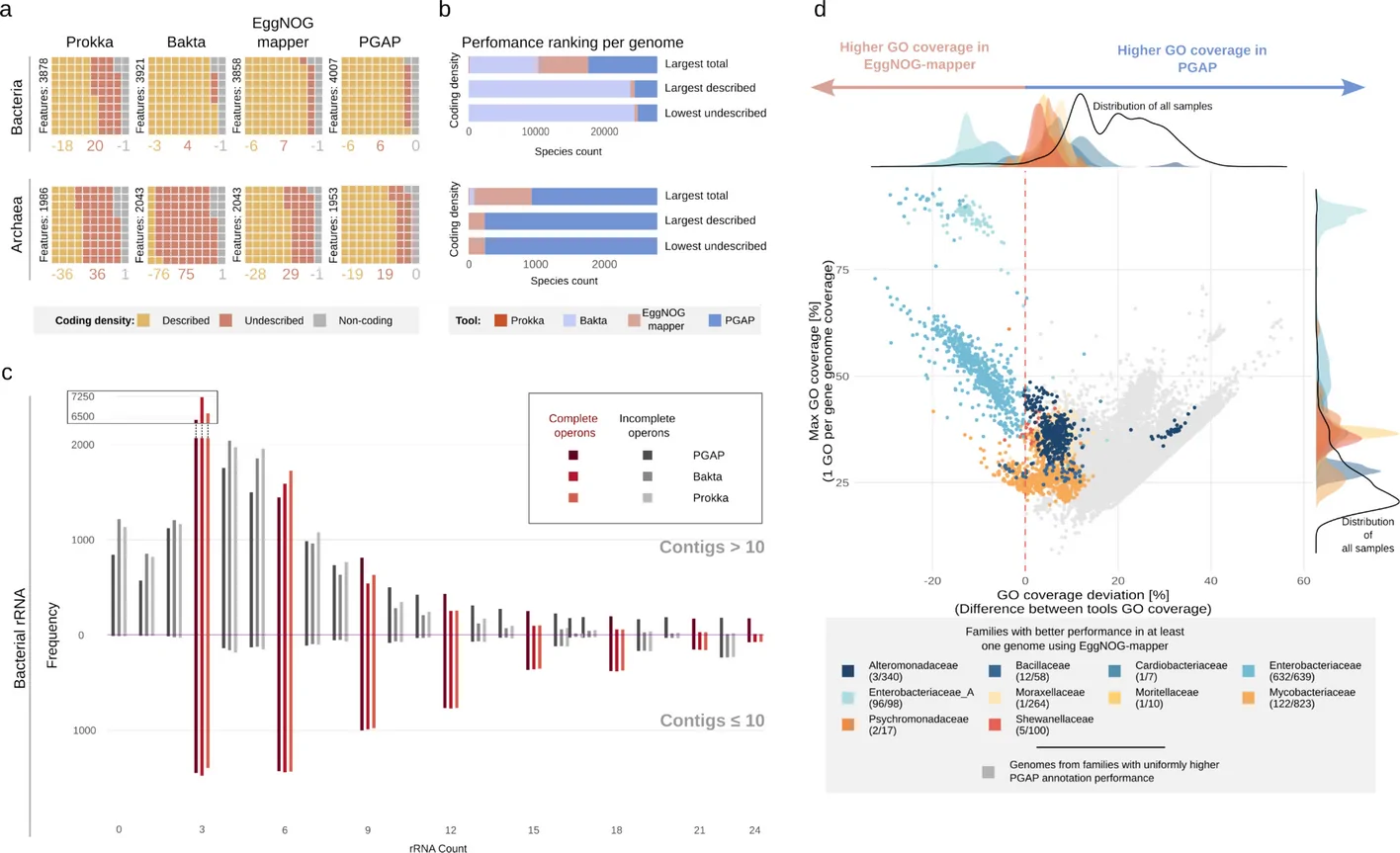
Taxonomy performance analysis. **a** Average coding density detected by each tool, partitioned into described (yellow), undescribed (red), and non-coding (gray) regions for Bacteria and Archaea. Each square represents 1% of the genome. Mean feature counts are shown on the left, with gains or losses relative to the E. coli baseline indicated below each plot. **b** Performance ranking per individual genome: Bar charts show how often each tool ranked best per species for total coding density, described coding density, and lowest undescribed coding density. Differences from the second-ranked tool were not considered. **c** Distribution of rRNA counts in complete and incomplete operons across bacterial genomes. The upper panel shows fragmented genomes (> 10 contigs), whereas the lower panel shows less fragmented genomes (< 10 contigs). Complete operons (multiples of three rRNA genes) are shown in red and incomplete operons in gray. Counts above 24 are omitted for clarity. **d** Comparison of Gene Ontology (GO) term coverage between PGAP and EggNOG-mapper across 26,970 bacterial genomes. Each point represents a genome; the y-axis shows the maximal GO coverage across both tools, and the x-axis the difference in coverage (EggNOG-mapper − PGAP). The red vertical line indicates equal coverage. The marginal density plot (black outline) shows the parameter distributions. Points right of the red line indicate higher PGAP coverage, whereas points left indicate higher EggNOG-mapper coverage. Gray dots denote genomes from families in which PGAP consistently outperformed EggNOG-mapper; colored dots indicate families with at least one genome favoring EggNOG-mapper. Legends report the number of such cases and total genomes per family. Enterobacteriaceae_A denotes a GTDB-recognized cluster with suffixed placeholder names [15, 16]

Coding density was analyzed within Archaea and Bacteria using the same metrics as the baseline: total, described, and undescribed coding density. The average total coding density across all tools ranged from 88.01% to 88.85% of genome length, aligning with the 87% reported in the literature [21]. While total coding density remained consistent with baseline performance, we observed a notable shift from described to undescribed coding density, particularly in Archaea (Fig. 3a). To provide “taxonomic-based” annotation recommendations, we identified the best-performing tool for each genome based on total and described coding density metrics (Fig. 3b). Detailed species-level results covering each tool's performance are available in Additional file 1: Fig. S12 and Additional file 2: Tables S3-4.

Total Coding Density: Among 26,970 bacterial species, PGAP annotated the largest total coding density for 9,870 species, followed by Bakta (9,852 species), EggNOG-mapper (7,169 species), and Prokka (79 species) (Fig. 3b, Additional file 1: Figs. S12-13a). In 2,791 archaeal genomes, PGAP showed the highest outcome in 1,858, EggNOG-mapper in 847 and Bakta in 72, Prokka in 8 (Fig. 3b, Additional file 1: Fig. S14a). These results suggest that no single tool consistently maximized total coding density across all taxa. While some genus-level performance trends were noted, no meaningful taxonomic recommendations could be made. Described Coding Density: Next, we checked how much of the total coding density is described. For bacteria, Bakta had the largest described coding density in 23,121 species, far outpacing PGAP (3,251 species) and EggNOG-mapper (596 species), with Prokka leading in 2 (Fig. 3b, Additional file 1: Fig. S13b, c). These results position Bakta as a strong general recommendation for annotating the described coding density in bacteria. In Archaea, PGAP led in 2,549 species and EggNOG-mapper in the remaining 242 species, while neither Bakta nor Prokka led in any cases (Fig. 3b, Archaea, Additional file 1: Fig. S14b, c). In bacteria, Bakta had the highest mean proportion of features with database accessions (UniRef50: 91.74%, UniRef100: 73.02%), followed by EggNOG-mapper (PFAM: 81.67%), PGAP (RefSeq: 67.69%), and Prokka (UniProtKB: 46.69%). Conversely, in Archaea, EggNOG-mapper showed the highest mean accession proportion (PFAM: 57.50%), with PGAP (RefSeq: 31.57%), Prokka (UniProtKB: 23.56%), and Bakta (UniRef50: 10.04%, UniRef100: 0.02%) following.

#### RNA features

The RNA section largely mirrors the structure of the baseline performance analysis. The results were similar in Bacteria, while in Archaea, these counts were lower across all tools (Additional file 1: Fig. S15, Additional file 2: Tables S3-4). To assess the correctness of the rRNA annotation, we investigated if they appear as full rRNA operon (multiples of 3 rRNA; Fig. 3c, red bars).

Among non-fragmented genomes (Contig ≤ 10), all tools exhibited high proportions of rRNA counts matching the complete operons (Bacteria: Bakta: 79.0%, PGAP: 78.5%, Prokka 77.5%; Archaea: Bakta: 67.2%, PGAP: 67.4%, Prokka 61.4%), which declined in highly fragmented genomes (Contig > 10; Bacteria: Bakta: 50.4%, PGAP: 49.3%, Prokka: 54.2%; Archaea: Bakta: 27.9%, PGAP: 35.4%, Prokka: 41.9%). For highly fragmented genomes, PGAP often predicted the highest number of rRNA sequences, suggesting its ability to identify all parts of a fragmented rRNA gene split across multiple contigs (Additional file 1: Figs. S15-16, Tables S9-11). In comparison to tRNA in the taxonomical variable group, Prokka and Bakta predicted a higher number of tRNAs (Prokka: mean = 54.27, SD = 18.84; Bakta: mean = 53.20, SD = 18.0) compared to PGAP (PGAP: mean = 51.6, SD = 17.5). Moreover, considering the minimum number of tRNAs required to cover 20 essential amino acids as proposed by Land et al. [21], Bakta failed to meet this requirement in fewer cases (352) than Prokka and PGAP (Prokka: 404, PGAP: 441). In Archaea, Bakta and PGAP produced consistent results (Bakta: mean = 38.54, SD = 10.58; PGAP: mean = 38.34, SD = 9.68; Prokka: mean = 30.13, SD = 14.45).

While bacterial tmRNA results were consistent with the baseline, we observed tmRNA sequences in 51 archaeal samples, where it is not known to occur. Following Nawrocki et al*.* [22], we attribute this to metagenome contamination. However, in 10 Bakta and 1 Prokka cases, it was found in non-metagenome samples (Additional file 2: Table S5).

#### Biological functional annotation

Building upon the preestablished biological function annotation baseline in *E. coli*, we observed a significant decline in Gene Ontology (GO) term coverage when extending the analysis beyond *E. coli* to Bacteria and Archaea. This decline was evident for annotations generated by Bakta and EggNOG-mapper.

In Bacteria, PGAP consistently provided the highest GO coverage (mean = 31.20%, SD = 6.92%), outperforming both EggNOG-mapper (mean = 11.44%, SD = 11.6%) and Bakta (mean = 2.40%, SD = 4.62%). Notably, Bakta did not exceed PGAP's GO coverage in any bacterial family. However, EggNOG-mapper performed better than PGAP in at least one genome in ten taxonomic families, including in a large portion of clinically relevant groups such as *Enterobacteriaceae*, *Mycobacteriaceae*, and *Bacillaceae* (Fig. 3d). In Archaea, the decline in GO coverage was even more pronounced. PGAP maintained the highest performance (mean = 20.03%, SD = 3.30%), while EggNOG-mapper (mean = 5.80%, SD = 4.44%) and Bakta (mean = 0.13%, SD = 0.11%) performed considerably worse. Across all tools, incorporating taxonomic variation reduced the average GO coverage, with PGAP remaining the most reliable option overall.

GO richness remained comparable to the baseline established in *E. coli,* in contrast to GO coverage. EggNOG-mapper demonstrated the highest mean GO richness in both Bacteria (mean = 35.40, SD = 10.60) and Archaea (mean = 38.69, SD = 14.30), far surpassing Bakta (Bacteria: mean = 3.56, SD = 1.18; Archaea: mean = 1.73, SD = 0.03) and PGAP (Bacteria: mean = 2.03, SD = 0.16; Archaea: mean = 2, SD = 0). This result reinforces EggNOG-mapper’s utility in generating richer functional annotations, albeit with the previously mentioned lower overall GO coverage compared to PGAP.

### Assessing annotation stability: metagenome-assembled genomes, frameshift sensitivity and the role of temporal delays in genome annotation performance

#### Metagenome-assembled genomes

Identifying novel microbes often relies on environmental samples and non- or hard-to-cultivable organisms, making isolates difficult to obtain. MAGs are, therefore, crucial; however, their correct assembly is complicated by factors such as strain mixtures, chimeric assemblies, incomplete sequences, truncated genes, and frameshifts [23]. We evaluated MAG annotation performance using 3,137 high-quality bacterial MAGs, all from different families (as defined by GTDB) to cover a broad taxonomic range. The selected MAGs exhibited lower completeness (mean = 85.22%, SD = 11.94%) and higher contamination levels (mean = 1.67%, SD = 1.59%) compared to non-MAGs (Additional file 1: Fig. S17). Archaeal MAGs were not investigated because they were included in the previous section due to the low number of non-MAGs.

**Fig. 4:**
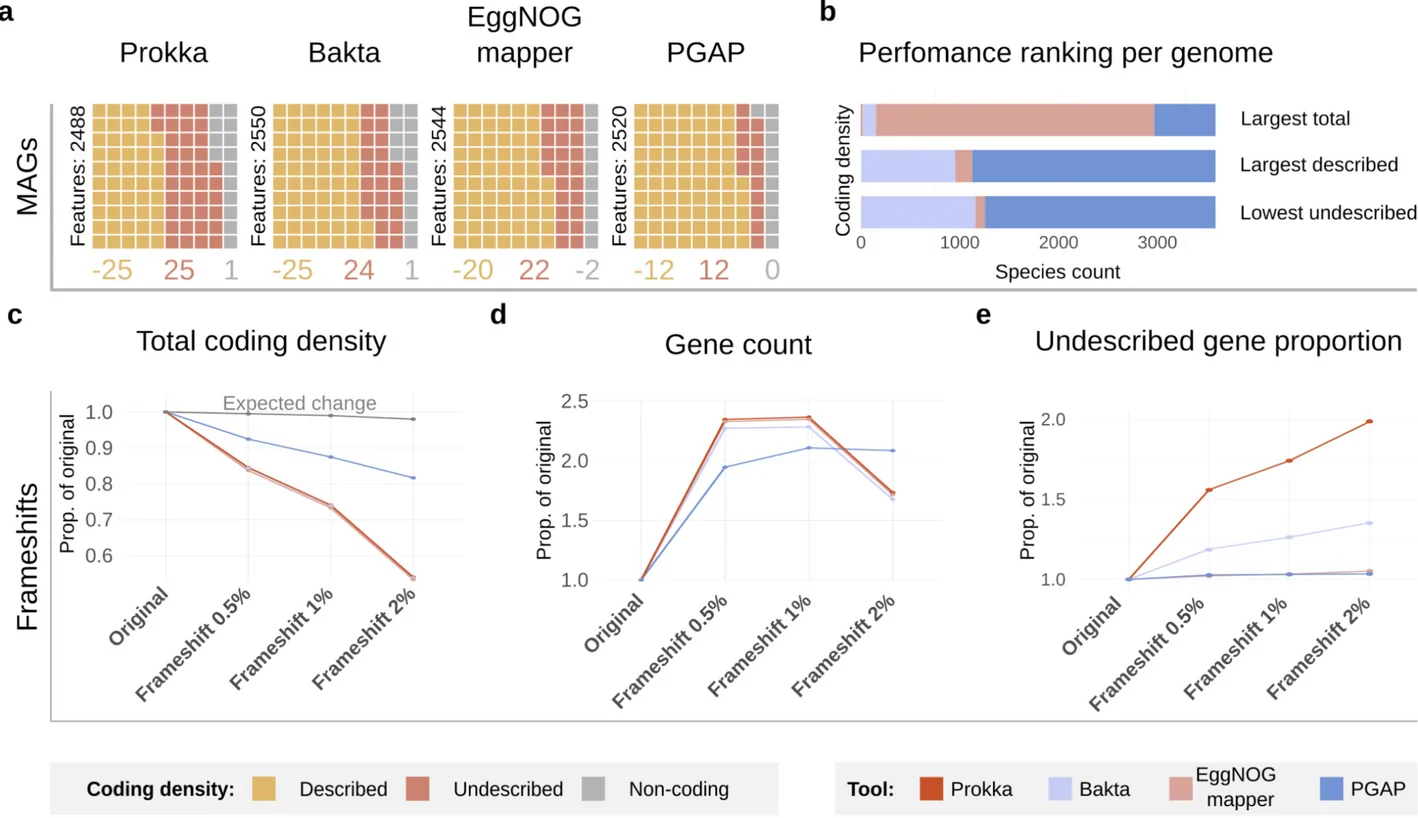
Impact of bacterial metagenome-assembled genomes (MAG)s and frameshifts on annotation quality. MAGs: **a** Breakdown of the average coding density detected by each tool, categorized into described coding density (yellow), undescribed coding density (red), and non-coding density (gray). Each square represents 1% of the total genome. The mean feature count is noted on the left. The annotated gain or loss to the E. coli baseline is outlined in colored numbers below each plot (e.g., a yellow −23 means that this tool annotated 23% less described coding density compared to the baseline). **b** Performance ranking per individual genome: The bar charts highlight the number of times a tool has achieved the best performance in one of the three categories for each species (largest total, largest described, and lowest undescribed coding density). This approach did not account for the performance difference between the best and second-best tools. Frameshifts: The effect of frameshifts on gene annotations was evaluated under three genome-wide deletion conditions (0.5%, 1%, and 2%) in Bacteria. We displayed mean proportion change to the original sequence of (**c**) retained coding density, **d** gene count, and **e** proportion of hypothetical features compared to the mean original value across four annotation tools (Prokka, Bakta, EggNOG-mapper, and PGAP). The total coding density plot (**c**) also includes expected differences in coding density based on the different deletion proportions

The comparison between MAGs and the previously established *E. coli* baseline revealed a substantial, yet non-uniform, shift from described to undescribed coding density, within each tool (Fig. 4a, b, Additional file 2: Table S6). This shift was more pronounced than the previously described taxonomic impact. Bakta experienced the most significant increase in undescribed coding density, whereas PGAP now provides the largest described and lowest undescribed coding density on average for MAGs. A substantial decrease in the described coding density was also observed in EggNOG-mapper, but to a lesser extent than Bakta. Additional gene length analysis is presented in Additional file 1: Figs. S18-19.

The rRNA measurements in MAGs generally followed the trends seen in the baseline measurements. PGAP identified more rRNA genes compared to other tools (PGAP: mean = 2.15, SD = 1.96; Prokka: mean = 1.66, SD = 1.55; Bakta: mean = 1.54, SD = 1.46). Regarding the biological functional annotation, all tools GO richness remained stable when compared to the baseline. The GO coverage of PGAP decreased to a similar level as the taxonomic variable group (mean = 31.54%, SD = 5.4%). In contrast, the decrease of other tools was higher (EggNOG-mapper: mean = 6.95%, SD = 5.28%; Bakta: mean = 0.18%, SD = 0.92%).

#### Frameshift sensitivity

During the processing of genome data, NCBI classifies samples as problematic if more than 30% of the protein-coding genes exhibit frameshifts (https://www.ncbi.nlm.nih.gov/datasets/docs/v2/policies-annotation/genome-processing/genome_notes/). We investigated the impact of frameshifts on annotation performance for each annotation tool by creating 98,742 frameshifted genomes. This subset was created by randomly deleting 0.5%, 1%, and 2% of nucleotides using 32,914 different genomes (Methods: Frameshifts). There are two aspects to consider for any given feature with a frameshift: first, the tool may fail to detect it or not fully annotate it, increasing the non-coding density; second, it may detect the sequence but split it into multiple features (increasing protein-coding gene count).

The total coding density decreases progressively as the deletion rate increases in all tools, but to a noticeably lesser extent in PGAP, compared to the original sequence (Fig. 4c).

First, the large decrease in total coding density for Prokka, Bakta, and EggNOG-mapper indicates that frameshifts are causing parts of features to be missed. Yet, there was a twofold increase in the total number of features from the original sequence to the 0.5% noise level, with feature count stability between 0.5% and 1%, followed by a decrease in feature count at 2% for all three tools (Fig. 4d). This suggests that these tools can detect certain split fragments of features but not all, leading to a gradual reduction in total coding density as noise increases while feature counts go up. However, at a 2% deletion rate, the feature count drops again, indicating that features become too broken up to be detected.

In contrast to the other three tools, PGAP exhibits a distinct pattern. The overall total coding density decreases less dramatically, and the feature count also increases to a lesser extent compared to the other tools. This combination of data suggests that PGAP is more effective at identifying most of the frameshifted parts while simultaneously annotating them into a single feature, thereby avoiding multiple feature descriptions for the same original feature (Additional file 1: Fig. S20, Additional file 2: Table S7). Additionally, the 2% noise rate did not result in a decrease in feature count, as previously observed in the other tools. Thus, PGAP is still able to annotate the fragmented features. Specifically, PGAP showed smaller feature increases at the 0.5% level, followed by slight increases at 1%, and stability at 2%, with low variability between samples (Fig. 4d). This demonstrates the benefit of using ORFfinder and ProSplign frameshift detection included in PGAP [6, 24], compared to the use of only Prodigal by Prokka and Bakta.

We found no significant shifts in rRNA and tRNA counts across different noise levels, likely due to the relatively small proportion of occupied coding density of these features and the requirement for splits to occur within these particular sequences.

#### Temporal impact

Annotation tools rely on databases like InterPro for gene function descriptions, which are regularly updated with new proteins (Additional file 1: Fig. S21). These database changes require the user to update their annotation tool to refresh their database (Table 1). We were able to evaluate this temporal impact on annotation quality, by comparing our current PGAP annotation feature count with older annotation information stored in the GTDB metadata (Additional file 1: Fig. S22). On average, our more recent PGAP annotations detected 139.8 (SD = 134.79) more features than the previous annotation, highlighting the importance of regular updates. As the exact time of the older annotation was not available, further analysis could not be performed based on the available data. There was no change in rRNA counts between the re-annotations.

## Discussion

The rapid growth in sequenced prokaryotic species and the discovery of new proteins underscore the importance of accurate and comprehensive genome annotation to enable meaningful biological downstream analysis. Given the diversity of prokaryotic microorganisms and the varying quality of genomic data, understanding the strengths and limitations of different annotation tools is paramount. Thus, we explored the capabilities and differences of various gene annotation tools designed for prokaryotic annotation, namely Prokka (19,000 citations [12]), Bakta (1,400 citations [13]), EggNOG-mapper (4,800 citations [14]), and PGAP (7,300 citations [6]), across a large and diverse dataset.

**Fig. 5:**
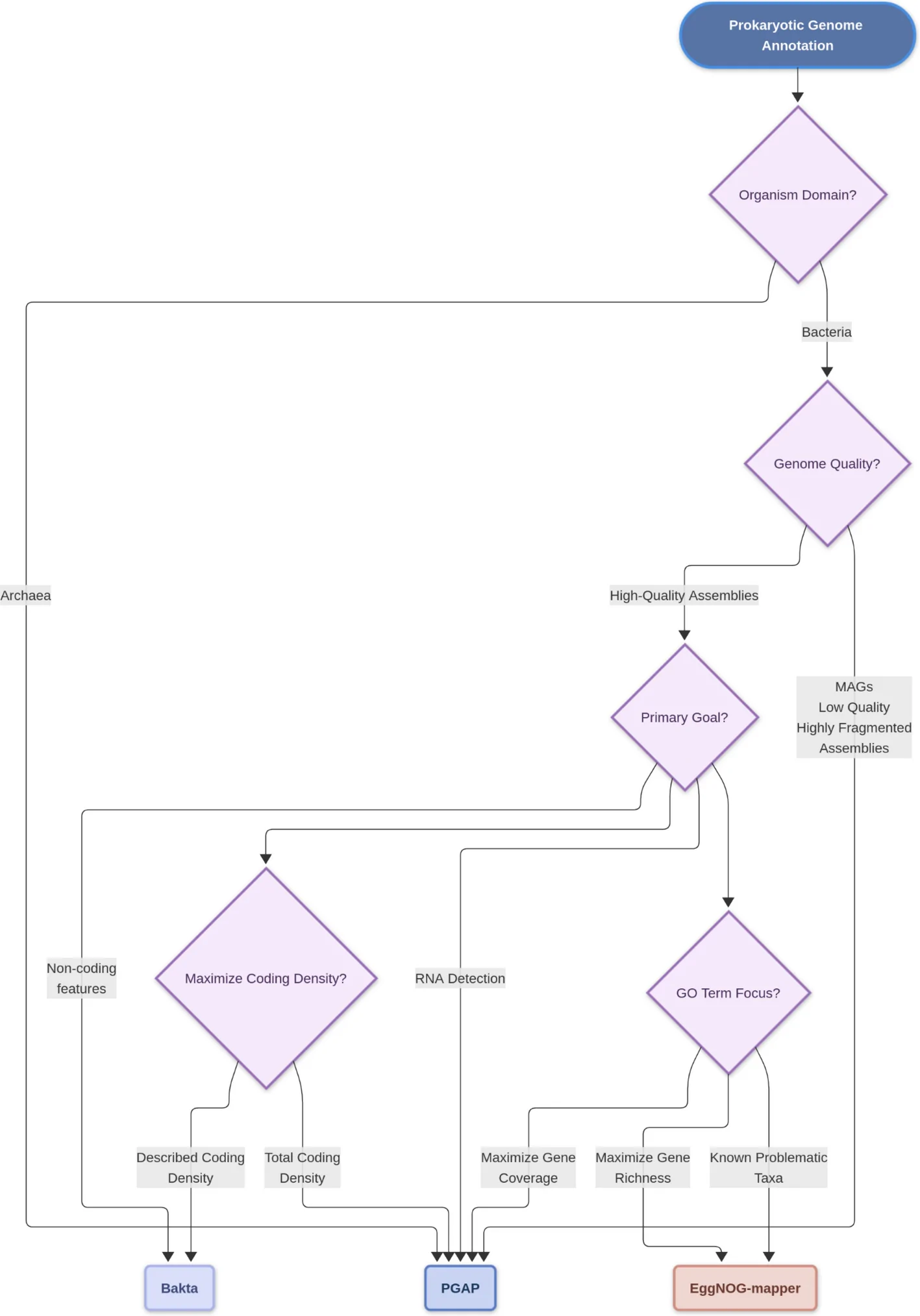
Simplified decision tree for selecting an appropriate annotation strategy. Prokka is generally not recommended; for projects with low resource requirements where feature detection is the priority, we recommend using Bakta light database as per the author of Prokka's recommendation. If functional annotation is required, the online versions of investigated tools are recommended

Even after analyzing and annotating over 156,033 genomes (including *E. coli* strains, bacteria, archaea, and simulated genomes), we did not observe a clear "winner" among the evaluated tools. Instead, our findings reveal specialized applications depending on genome properties and research objectives. This supports a "choose the right tool for the right genome and goal" approach, rather than a “one-size-fits-all” strategy. We thus outline recommendations based on genome characteristics and annotation priorities (Fig. 5).

For typical bacterial isolates, our analysis revealed a nuanced tool recommendation between Bakta and PGAP. While taxonomic variation did not lead to clear genus-level recommendations for maximizing total coding density, Bakta consistently annotated the largest described coding density across many bacterial species. This suggests Bakta is highly effective at assigning known functional descriptions to coding sequences in bacterial isolates. PGAP, on the other hand, annotated the largest total coding density in a similar number of bacterial species and demonstrated reliable rRNA, tRNA, and tmRNA detection in fragmented bacterial genomes. Therefore, Bakta is strongly recommended for bacterial isolates when maximizing the described coding density, which is the primary goal. In contrast, PGAP is recommended for maximizing total coding density in fragmented genomes e.g. from short-read assembly or ensuring robust detection of RNA features.

In Archaea, our results indicate a more apparent preference for PGAP. Across 2,791 archaeal genomes (including metagenome-assembled genomes (MAGs)), PGAP consistently annotated the largest total and described coding density, significantly outpacing other tools. PGAP also performed better in detecting rRNA in more fragmented archaeal genomes. This suggests PGAP is the most robust option for archaeal annotation, regardless of whether the genome is an isolate or a MAG.

The annotation of "problematic genomes," such as MAGs or those with significant fragmentation, errors, or potential contamination, presents unique challenges. PGAP showed the best stability in MAG annotation, by providing the largest described coding density and the lowest undescribed coding density on average. Furthermore, our targeted frameshift analysis, simulating problematic sequencing errors, revealed that PGAP is significantly more resilient to sequence errors, maintaining a more consistent annotated coding density and feature count compared to Bakta, EggNOG-mapper, and Prokka, which showed greater disruptions. Considering these factors, PGAP is a strongly recommended tool for annotating potentially problematic genomes, where issues such as fragmentation, contamination, and sequencing errors are more likely to occur.

Biological function assignment depends on prior feature detection, thus results must be interpreted considering the total coding density. The Gene Ontology (GO) term assignment varied between the samples but PGAP and EggNOG-mapper stand out as clear recommendations. PGAP consistently provided the highest GO coverage (the proportion of genes with any GO term assigned) across Bacteria and Archaea, including MAGs, albeit with lower GO richness (the number of unique terms per annotated gene) than EggNOG-mapper. However, EggNOG-mapper showed better GO coverage than PGAP in specific bacterial families, including some with clinical relevance, which should be considered when choosing. Therefore, the choice for biological function annotation depends on the particular goal. If maximizing the number of genes with any function assignment is critical (coverage), PGAP is generally recommended, especially for diverse or lower-quality genomes. If obtaining a wider variety of GO terms for annotated genes is prioritized (richness), EggNOG-mapper performs better, particularly in less problematic genomes and specific taxa. In many cases, users might consider running both tools complementarily. Moreover, by manually investigating assigned protein database accessions (e.g., UniRef, UniProtKB), users can access additional functional information and controlled vocabulary terms extending beyond the core annotation workflow databases.

While EggNOG-mapper is highly effective for functional assignment, it lacks the structural feature-prediction refinement found in comprehensive workflows, such as the active filtering of known false positives. Consequently, it may retain spurious predictions that other tools would otherwise exclude.

The practical deployment of these workflows is strictly bounded by their database sizes and hardware footprints. As shown in Table 1, significant disparities exist in storage requirements: Bakta and EggNOG-mapper maintain the largest databases (exceeding 50 GB), whereas Prokka utilizes the smallest database and hardware footprint (under 1 GB). Prokka thus represents a highly efficient, yet functionally limited alternative in low-resource computing environments where deploying a purely feature-detection solution like Prodigal or utilizing online web servers is impossible.

Furthermore, runtime processing demands a careful balance of system resources. PGAP exhibits the highest CPU time requirements by a substantial margin, making large-scale local deployments computationally heavy. Meanwhile, EggNOG-mapper offers a high-memory mode (–dbmem) designed for rapid database caching to optimize speed, but this introduces a strict hardware bottleneck by demanding 44 to 48 GB of RAM.

If working in a hardware-constrained environment, researchers can leverage the web-based versions of tools like EggNOG-mapper or Bakta, which offer comparable annotation quality to local executions. However, if the annotation must be performed locally under strict resource constraints, users may opt for Prokka or Bakta with its "db-light" database parameter, though they should remain mindful of the reduced performance and annotation depth associated with these configurations. Ultimately, users must carefully weigh these infrastructural constraints alongside their available hardware and taxonomic focus when selecting the optimal annotation tool.

Looking forward, the next generation of prokaryotic annotators must transition from isolated feature-finding pipelines toward unified, comparative frameworks that improve functional predictions for undescribed features. Ideally, these platforms should allow for the integrated use of multiple distinct annotation tools within a single analysis, akin to third-party solutions like Metannotator [25], by providing machine-parsable unified output understood by 3rd party tools. When sequence-homology approaches fail, integrating deep-learning structural predictors (such as AlphaFold and its successors) into standard pipelines will be critical for mapping three-dimensional protein folds directly to functional categories.

Furthermore, next-generation tools must expand their scope beyond coding sequences to reliably model entire transcriptional units. While pipelines like Bakta have begun tracking specific non-coding elements, future platforms must integrate automated predictions of complex regulatory architectures. This means seamlessly unifying accurate start codon assignments, promoters, ribosomal binding sites (RBS), and transcriptional terminators.

Finally, tool developers must focus on streamlining the interpretation of downstream results. Future workflows should transition away from flat output files toward standardized, semantically queryable data structures. This shift will allow researchers to effortlessly filter, search, and isolate specific genomic features or biological systems of interest.

## Conclusions

Our comprehensive evaluation of prokaryotic genome annotation tools highlights that no single tool is universally optimal. The ideal choice is contingent upon several factors, including the taxonomic affiliation of the organism, the quality and fragmentation of the genome assembly (e.g., isolates vs. MAGs, presence of errors), and the specific research goal (e.g., maximizing total coding density, maximizing described function, reliable RNA annotation). Our research emphasizes the importance of considering taxonomy, sequencing technologies, sample quality, and database updates when using, benchmarking, or developing annotation tools. Understanding these characteristics is essential for both tool users, who must select the most appropriate tool for their specific data and question, and developers, who can use these insights to improve future algorithms and database integration. Therefore, this work offers valuable insights for both groups by demonstrating overall patterns in tool behavior and potential problem areas, which will continue to be relevant in the future for prokaryotic genomics.

## Methods

### Datasets

#### Data querying and extraction

All analyzed prokaryotic genomes were obtained by querying the release 207.0 of Genome Taxonomy Database (GTDB) (https://gtdb.ecogenomic.org), contained in the release files bac120_metadata_r207.tsv for Bacteria and ar53_metadata_r207.tsv for Archaea records. GTDB files were also used for sample metadata. Genome assemblies were then downloaded from NCBI Datasets repository via. Next, the samples were rehydrated with “datasets rehydrate –directory ncbi_dataset”. The quality of samples was measured by inspecting NCBI-reported CheckM completeness, contamination, and count of consensus-assembled genomic regions (contigs) (Additional file 1: Figs. S4, 8–11). The failure to download and analyze 4,660 samples (Additional file 1: Fig. S1) was primarily due to obsolete genomes in the NCBI database, taxonomic assignment errors that caused annotation failures, or other technical issues. 

Please note that the choice of reference database and taxonomy impacts the annotation results. We decided to adhere to the taxonomy classification provided by GTDB and their more stringent taxonomy verification schema, which we treat as a gold standard for this work. For additional dataset details, please refer to the GTDB release 207 website. However, biological databases are continuously updated as new bacterial species are discovered, particularly in the context of metagenome-assembled genomes (MAGs) and environmental samples. For example, within the year 2021, 12,556 new candidate species-level operational taxonomic units (OTUs) were identified [26]. The GTDB database also experienced a significant increase, adding 17,809 Bacteria and Archaea species between release-202 in 2021 and release-207 in 2022 (Additional file 1: Fig. S23). Additionally, the taxonomic assignments for each genome are registered between GTDB releases, which allows for a history of assignments across newer and older releases.

#### Escherichia coli

*E. coli* samples were derived by extracting part of the string that starts with “s__” from the database key “gtdb_taxonomy” with command “str_extract(bac120_r207$gtdb_taxonomy”, "s__[a-zA-Z\\s] + ") from string R library. The output was queried for “s__Escherichia coli”. 

The analysis included 24,393 *E. coli* samples, with an average completeness of 99.72% (SD = 1.11%) and contamination levels averaging 0.41% (SD = 0.69%). The mean number of contigs per assembly was 185.04 (SD = 149.54), with a genome size averaging 5,120,392 base pairs (SD = 274,048 bp). Variations in genome size among *E. coli* samples likely result from assembly issues, samples derived from metagenomes, or natural intraspecies variability [27]. From the 24,393 genomes analyzed, we identified 2,073 distinct serotypes based on their O- and H-types in 20,525 genomes, where in 10 samples serotyping failed. Among the remaining genomes, 3,336 had only an H-type, 358 had only an O-type, and 164 had neither (Additional file 2: Table S1).

#### Archaea and bacteria

The representative group for both Bacteria and Archaea files was queried with database key “gtdb_representatitive” for value “t” for GTDB representative genomes. 

The dataset comprised 30,107 Bacteria and 2,791 Archaea samples (Additional file 1: Figs. S2-3). Bacteria showed an average completeness of 95.45% (SD = 9.12%), while Archaea had a completeness of 85.57% (SD = 13.02%). Contamination levels averaged 1.03% (SD = 1.34%) for Bacteria and 1.28% (SD = 1.58%) for Archaea. The mean number of contigs per assembly was 93.38 (SD = 154.05) for Bacteria and 113.9 (SD = 128.18) for Archaea. Genome sizes averaged 4,009,159 bp (SD = 2,120,640 bp) for Bacteria and 1,895,897 bp (SD = 1,042,641 bp) for Archaea. Detailed dataset information is available in Additional file 1: Figs. S8-11.

Overall, our preliminary dataset statistics indicate that the genome assemblies were sourced from high-quality samples, with minimal potential contamination, making them suitable for downstream analysis.

#### Frameshifts

To simulate genomes of lower assembly quality and the effects of open reading frame shifts on annotation quality, we modified the obtained FASTA files of the samples by inserting pseudo-random deletions. In the analysis, we use 0.5%, 1%, and 2% deletions of nucleotides in a sample’s FASTA file. The frameshift analysis was performed on a 32,914-genome subset of archaea, bacterial and *Escherichia coli* samples, generating in total 98,742 data points. Considering the assumption of equal spread of deletion events and an average bacterial genome length of 4,372,147 bp in our dataset, we anticipate deletion occurrences at regular intervals of 199, 99, and 49 bp, respectively.

The expected fragment length between deletions was calculated via

$$Deleted\;Nucleotides =\frac{Percentage\;Deleted}{100}* Original\;Genome\;Length$$

$$Number\;of\;Fragments = \frac{Original\;Genome\;Length- Deleted\;Nucleotides}{Deleted\;Nucleotides}+1$$

$$Average\;Fragment\;Length =\frac{Total\;Remaining\;Genome\;Length }{Number\;of\;Fragments}$$

The random deletions are generated with the following code stored in https://github.com/AggresiveHayBale/anno_bench in script “noise.sh”.

#### Metagenome-assembled genomes

We recognize a genome as a metagenome if the key “*ncbi_genome_category”* has a value “*derived from metagenome*". The dataset comprises 101 samples designated as metagenome-assembled genomes in the *E. coli* group, 3,137 in the bacterial group, and 2,031 in Archaea. The *E. coli* and Archaea metagenomic samples were excluded from metagenome analysis due to low counts (*E. coli*) and significant differences in tool performance compared to the other groups (Archaea).

#### Additional parameters

The NCBI submission date was extracted from the key “*ncbi_date*” located in the dataset file. The used dataset consists of samples submitted between 2000 and 2021 (Additional file 1: Fig. S22). The NCBI-reported protein count is derived from the key “*ncbi_protein_count*” while PGAP annotation is the total count of CDS from de novo annotation done for this benchmark.

### Annotation and tool description

We recognize that specialized single-function tools, that depend on previously detected feature starts and ends, like DEEP-FRI [28], may outperform generalist tools in specific tasks.These tools were excluded from this analysis due to their specialized focus on either a singular step of describing previously detected features or the annotation of specific subsets of features. Furthermore, popular tools like RAST [29], which rely on external servers, were excluded from our benchmark due to technical limitations imposed by the use of external, not self-managed servers, thereby limiting the potential for a large-scale benchmark. The selected tools use a varied approach to CDS feature prediction using Prodigal (Prokka, EggNOG-mapper), Prodigal with improved short ORF detection (Bakta), and ORFfinder with a large set of other tools (PGAP). For a detailed description of each tool, please refer to the original articles.

### Tools

Tools were used with the suggested default parameters, as outlined in the respective GitHub help pages. In the case of EggNOG-mapper, the parameters of the default setting for the online version of the tool (http://eggnog-mapper.embl.de) were used for the “Genome” data type and Gene prediction method “Prodigal” and “Diamond” search method. Additional files 2: Tables S8-11 consists of data about performance of individual samples in each taxonomic rank across investigated parameters.

### Genetic code translation table

For Mycoplasmatota (formerly Tenericutes), the 4th translation table was used instead of the standard one [30]. Using flags:

- Prokka: –gcode 4
- Bakta: –translation-table 4
- EggNOG-mapper: –trans_table 4
- PGAP does not require a flag as it chooses the translational table using the species name to make a decision

#### Prokka

Containerized Prokka 1.15.6 (DockerHub container: staphb/prokka:1.15.6) was executed with command:

- prokka –compliant –outdir output –force –prefix ${name}_prokka –quiet ${fasta}

The Prokka database used for the annotation is specific to the Prokka version and is an integral part of its GitHub repository. Prokka and its bundled database were the oldest of all the investigated tools, with the last update in November 2019 (v1.14.5; https://github.com/tseemann/prokka/releases). This indicates that the functional annotations provided by Prokka may not be as current as those offered by other tools. Prokka is a popular, fast, and lightweight tool suitable for both small-scale system requirements and low storage needs (database size: 0.6 GB), developed in 2014 by Torsten Seemann for draft genome annotations. Prokka employs as its core databases ISfinder, NCBI Bacterial Antimicrobial Resistance Reference Gene Database, and UniProtKB (SwissProt), followed by bacteria-specific HMM libraries such as Pfam and TIGRFAMs for annotation of predicted CDS features [12].

### Bakta

Containerized Bakta 1.7 (Docker Hub container: nanozoo/bakta:1.7.0–23ae510) was executed with command:

- bakta –prefix ${name}_bakta –db database/–keep-contig-headers ${fasta}

The annotation was executed with the database version 5.0.

Bakta, while taking inspiration from Prokka, offers more advanced taxon-independent annotation capabilities while retaining Prokka's user-friendly simplicity. Bakta had 40 releases between November 2020 and July 2024 (v1.9.4; https://github.com/oschwengers/bakta/releases), while its database had 8 releases (https://zenodo.org/records/10522951), with the most recent on January 19, 2024 (v5.1). Bakta requires a larger database than Prokka (60 GB) and has higher hardware requirements. It also provides a web frontend for small-scale annotation. Bakta annotates features such as tRNA, tmRNA, rRNA, ncRNA genes, CRISPR, CDS, pseudogenes, ncRNA cis-regulatory regions, oriC/oriV/oriT, and assembly gaps. The database used for annotation incorporates UniProt's UniRef100 clusters, UniParc, and NCBI RefSeq sequences, with UniProt's UniRef90 seed sequences as a fallback. Bakta also integrates external databases such as NCBI AMRFinderPlus, COG, DoriC, ISFinder, Mob-suite, Pfam, Rfam, RefSeq, UniProtKB/Swiss-Prot, and VFDB. Functional annotation in Bakta includes KEGG pathways, PFAM protein families, RFAM RNA families, enzyme commission numbers, Clusters of Orthologous Groups, and Sequence Ontology terms.

#### EggNOG-mapper

Containerized EggNOG-mapper 2.1.9 (Docker Hub container: nanozoo/eggnog-mapper:2.1.9–4f2b6c0) was executed with command:

- emapper.py -i ${fasta} -m diamond –data_dir ${eggnog_db_dir} –itype genome –genepred prodigal –dmnd_ignore_warnings –dbmem –translate –go_evidence non-electronic –pfam_realign none –report_orthologs –decorate_gff yes –evalue 0.001 –score 60 –pident 40 –query_cover 20 –subject_cover 20 –tax_scope auto –target_orthologs all -o ${name}_eggnog

This evaluation adhered to EggNOG-mapper's default recommended settings, which inherently limited its Gene Ontology (GO) term annotation capabilities to a curated dataset of non-electronic GO terms (https://github.com/eggnogdb/eggnog-mapper/wiki/eggNOG-mapper-v2.1.5-to-v2.1.13#user-content-Basic_usage, Accessed on 17 November 2025). This imposed a restriction on EggNOG-mapper that other tools, not bound by these specific default settings, did not face.

We used the respective version of the database from November 22, 2022.

EggNOG-mapper is unique in its focus on orthology, allowing for the annotation of both prokaryotes and eukaryotes, unlike other tools that target only prokaryotes and employ a homology approach. Similar to Bakta, it provides a web frontend for small-scale annotation. EggNOG-mapper had 19 releases from November 2017 to August 2023 (v2.1.12; https://github.com/eggnogdb/eggnog-mapper/releases). EggNOG-mapper requires a similar large database to Bakta (51 GB). Its database had 11 releases, but the latest (v6.0.0) is not implemented in the tool available on GitHub, which uses version 5.0.2 from March 2021. It exclusively annotates gene type features, excluding any other type (rRNA, tRNA). EggNOG-mapper annotations are derived from their internal EggNOG database [31]. The functional annotation by EggNOG-mapper includes categories such as CAZy, COG, COG Categories, Enzyme Commission numbers, KEGG orthologs, BRITE hierarchies, BiGG Reactions, KEGG reaction classes, KEGG transporter classifications, KEGG pathways, KEGG reactions, KEGG modules, and PFAMs.

### PGAP

Containerized PGAP 2022–10-03.build 6384 (Docker Hub container: ncbi/pgap:2022–10-03.build6384) was executed with command:

- cwltool/pgap/pgap/pgap.cwl –fasta ${fasta} –ignore_all_errors –report_usage –submol meta.yaml

With the respective version of the database accessed on February 7, 2023. The taxonomic information (species name) in the “meta.yaml” file is derived from the GTDB database “ncbi_taxonomy” key.

PGAP has been in constant development by NCBI since 2001 and is internally used for annotating NCBI databases. PGAP had 31 releases from July 2018 to July 2024. Before the GitHub releases, updates were announced on the NCBI website, with 20 versions released between 2013 and 2018 (https://www.ncbi.nlm.nih.gov/refseq/annotation_prok/release_notes/). PGAP has a smaller database compared to Bakta and EggNOG-mapper, however larger than Prokka (30.4 GB). Since PGAP updates its database with each release, it is challenging to determine the exact number of database updates that have occurred. However, manual inspection revealed that 14 GitHub releases mentioned database changes, with an average interval of 123 days, making PGAP the tool with the most frequent database updates. The main distinguishing feature is the requirement of the tool for taxonomic information, tailoring the annotations to genome-specific patterns, and reducing the risk of human errors (wrong translational table use, detection of contamination, or wrong species assignment). The primary drawback of the tool is its relatively high computational requirements and the need for metadata preparation. PGAP distinguishes the following types of features: Gene, CDS, rRNA, tRNA, ncRNA, and CRISPR. It utilizes multiple data sources like TIGRFAM, Pfam, Rfam, PRK, NCBIfams, BlastRules, and several in-house created databases.

#### Workflow execution

The tools were run in a managed computing service environment on the Google Cloud Platform infrastructure, using the Google Cloud Batch executor. This was accomplished through a custom-built Nextflow [32] pipeline (https://github.com/AggresiveHayBale/anno_bench) and Docker containers (Additional file 1: Fig. S24). The virtual hardware specifications used for tool execution are as follows:

1. Prokka
  Docker Hub container: nanozoo/prokka:1.14.6–c99ff65
  CPUs: 4
  Memory: 2 GB
2. Bakta
  Docker Hub container: nanozoo/bakta:1.7.0–23ae510
  CPUs: 8
  Memory: 14 GB
3. EggNOG-mapper
  Docker Hub container:: nanozoo/eggnog-mapper:2.1.9–4f2b6c0
  CPUs: 6
  Memory: 48 GB
4. PGAP
  Docker Hub container: ncbi/pgap:2022–10-03.build6384
  CPUs: 8
  Memory: 8 GB

The median RAM utilization (Physical memory usage) and median CPU time (Task execution real-time) were read from the Nextflow execution report.

## Data analysis

Parameter list

1. General Annotation Statistics
  Gene count, Undescribed genes count, rRNA, tRNA, and tmRNA counts, Domain Unknown Function Count, Total Coding Space, Described Coding Space, and Undescribed Coding Space
2. Average feature length
  All Feature Length, Described Feature Length, and Undescribed Feature Length
3. Gene Ontology (GO) Metrics
  GO Coverage, GO Coverage in Described Features, GO Richness, GO Molecular Function, GO Biological Process, GO Cellular Component
4. Functional Database Annotation Countsand Proportion
  COG, Prodigal, UniprotKB, EC KEGG, PFAM, RFAM COG Group, SO UniParc, UniRef100, UniRef90, UniRef50, CAZy, COG Cat KEGG ko, BRITE, BiGG Reaction KEGG rclass, KEGG TC, KEGG Pathway KEGG Reaction, KEGG Module, PFAMs RefSeq

In contrast to similar studies [11], we focus on the measurement of coding density. Coding density denotes the proportion of the genome containing features in comparison to the genome size. We distinguish between coding density and coding space that denotes the raw sum of lengths of all detected features. Features length is defined by subtracting the stop position from the start position in the annotation file. The described coding density denotes the coding space of all features with feature description, recognized by a string starting with “product = ”(Prokka, PGAP),”Name = ” (Bakta), “em_desc = ” (EggNOG-mapper), and ending with “;” in all of the tools. The proportion of the above-mentioned coding densities is a proportion of the relative category relative to the total genome size reported by the NCBI in the metadata field “ncbi_genome_size”.

$$Gene\,Length (L)=Stop\;Position - Start\;Position$$

$$Total\;Coding\;Space = \sum\limits_{i}\,{L}_{i}$$

$$Described\;Coding\;Space = \sum\limits_{i-j}\,{L}_{i-j}$$

$$Undescribed\;Coding\;Space = \sum\limits_{j}\,{L}_{j}$$

$$Coding\;Density = \frac{{\sum}_{i}\,{L}_{i}}{NCBI Genome Size}$$

where i denotes the feature count and j denotes the undescribed feature count.

We prefer coding density metric over the total feature count to minimize the risk of artificially inflated or reduced gene counts stemming from differences in how tools merge and split subunits of genes or report only fragments of a complete feature. This approach helps mitigate such biases. However, due to the limited coding density and the conservative nature of RNA subgroups, we use the raw count for comparing these types of features.

When the undescribed feature proportion is used, the proportion of hypothetical proteins is calculated as follows:

$$Proportion\;of\;Undescribed\;Proteins\;(\%) = \frac{{\sum }Undescribed\;feature\;count* 100\%}{N}$$

where N denotes the overall feature count.

GO coverage and richness were calculated in the following manner:

$$GO\;coverage (\%) = \frac{{\sum }Features\;with\;at\;least\;one\;GO\;term * 100\%}{N}$$

$$GO\;richness = \frac{{\sum }GO\;terms\;per\;gene}{N}$$

where N denotes the overall feature count.

The original and modified sequences (frameshifts) are then annotated with Prokka, Bakta, EggNOG-mapper, and PGAP. The created annotation files in.gff3 (Bakta) and.gff (Prokka, EggNOG-mapper, PGAP) format are passed to the R language script that performs an automated count of the features and preliminary analysis.

Annotations were analyzed with R [33] (4.2.1) with Tidyverse [34] packages (tidyr (1.2.1), dplyr (1.0.10), readr (2.1.3), and stringr (1.4.1)). Additional data visualization was performed using following R libraries: viridis(0.6.3), waffle(0.7.0), hrbrthemes(0.8.6), RColorBrewer (1.1–3), ggpubr(0.6.0), gridExtra (2.3), ggplot2 [34] (3.4.2), nvimcom (0.9–147), and geneviewer (0.1.11).

The features were treated as undescribed if Prokka, Bakta, and PGAP described them as “hypothetical protein”, or in the case of EggNOG-mapper, if the product does not have a description (denoted as “None” or “NA”).

RNA features are recognized by extracting the “rRNA”, “tmRNA”, and “tRNA” from the 3rd column of the main annotation file (.gff,.gff3) in all of the tools.

GO terms and other functional annotation terms were extracted by querying the 9th column of the main annotation file, in case of

Prokka by: “GO”, “COG[0–9]{1,4}”.

Bakta: "GO:", "KEGG:", "PFAM:", "RFAM:", "EC:", "COG:COG", "COG:(?!COG)" (COG group), "SO:"

EggNOG-mapper The extracted terms are: "em_CAZy", "em_COG_cat", "em_EC", "em_KEGG_ko", "em_BRITE", "em_BiGG_Reaction", "em_KEGG_rclass", “em_KEGG_TC", "em_KEGG_Pathway", "em_KEGG_Reaction", “em_KEGG_Module", "em_PFAMs".

PGAP: “GO:”

The proportion of proteins with at least one GO term was calculated via

$$Term\;proportion =\frac{{F}_{x\ge 0} }{i}* 100$$

where *F* denotes features, *x* is the count of terms per feature, and *i* is the total feature count.

The serotyping was done using ECTyper [35] (1.0.0) using the command:

- ecoli_serotyper.py -i ${fasta}

## Supplementary Information

Additional file 1: Supplementary figures and tables. This file contains Supplementary Figuresand Tablesproviding the dataset taxonomy distribution, descriptive sample analysis, additional tool comparisons, frameshift and temporal analyses, GO-term analysis, and workflow visualization

Additional file 2: Data spreadsheets. This file includes a list of analyzed samples and summary statisticsfor the following groups: *Escherichia coli*, Archaea, non-MAG Bacteria, and MAG Bacteria. It also contains frameshifted genome data, best-performing tools with all investigated in this study genome accession numbers for *E. coli*, Archaea, non-MAG Bacteria, and MAG Bacteria, as well as accession lists for Archaeal samples with detected tmRNAand *E. coli* serotypesgenerated by the study workflow.

## Data Availability

The data used for benchmarking are available in release 207.0 of the Genome Taxonomy Database [15, 16, 36] (https://gtdb.ecogenomic.org) with corresponding NCBI Genbank [37, 38] accession identifiers. Additional metadata for the samples were derived from the Integrated Microbial Genomes and Microbiomes (IMG/M) [39] https://img.jgi.doe.gov database, accessed on April 28, 2023, and merged via the aforementioned identifier to the sample information. Analyzed sample list in the Additional file 2: Table S.6–9 in “NCBI Accession” data column and workflow output are deposited in Zenodo repository [40] (https://doi.org/10.5281/zenodo.21838357) under Creative Commons Attribution 4.0 International license. The script used for evaluation is available in a public GitHub repository [41] and Zenodo repository [42] (https://doi.org/10.5281/zenodo.20632848) under GPL-3.0 license.
