# Supplementary material for: Large-scale benchmarking of prokaryotic annotation tools across thousands of species
Source: Genome Biol. 2026 Sep 4;27:284. doi: 10.1186/s13059-026-04262-0 (PMC13548610; doi:10.1186/s13059-026-04262-0)
Supplement: Supplementary file 1 [file 13059_2026_4262_MOESM1_ESM.pdf]

Additional file 1

Contents

1 Dataset selection process for the Bacteria and Archaea kingdoms, *E. coli*, and MAGs 2

2 Dataset composition 3

3 Baseline annotation (*E. coli*) 5
- 3.1 Data description for *E. coli* strains . . . 5
- 3.2 Differences in assembly-gap annotation between tools . . . 6
- 3.3 Total feature count . . . 7
- 3.4 Undescribed feature count . . . 7
- 3.5 Total feature length . . . 7
- 3.6 Undescribed feature length . . . 7
- 3.7 Total annotated coding space . . . 8
- 3.8 Described coding space . . . 8
- 3.9 Undescribed coding space . . . 8
- 3.10 Impact on rRNA and tRNA discoverability in *E. coli* . . . 9

4 Quality analysis of GO terms in *Escherichia coli* K-12 substr. MDS42 10

5 The Bacteria and Archaea kingdoms 11
- 5.1 Data description for the Bacteria kingdom . . . 11
- 5.2 Data description for the Archaea kingdom . . . 12
- 5.3 Total coding density in the Bacteria kingdom . . . 13
- 5.4 Feature length comparison in the Bacteria kingdom . . . 14
- 5.5 Feature length comparison in the Archaea kingdom . . . 15
- 5.6 rRNA comparison for the Archaea kingdom . . . 16
- 5.7 Analysis of rRNA prediction variance in the Bacteria kingdom . . . 17
- 5.8 Capability of PGAP to annotate rRNA features in genomes with high contig counts . . . 20

6 Metagenome-assembled genomes (MAGs) 21
- 6.1 Data description . . . 21
- 6.2 Coding-length comparison . . . 22

7 Frameshifted genomes 23
- 7.1 Genome region comparison . . . 23

8 Temporal analysis 24
- 8.1 Annotation performance over time . . . 25
- 8.2 Increase in unique species in the database over time . . . 26

9 Workflow visualization 27

1 Dataset selection process for the Bacteria and Archaea kingdoms, *E. coli*, and MAGs

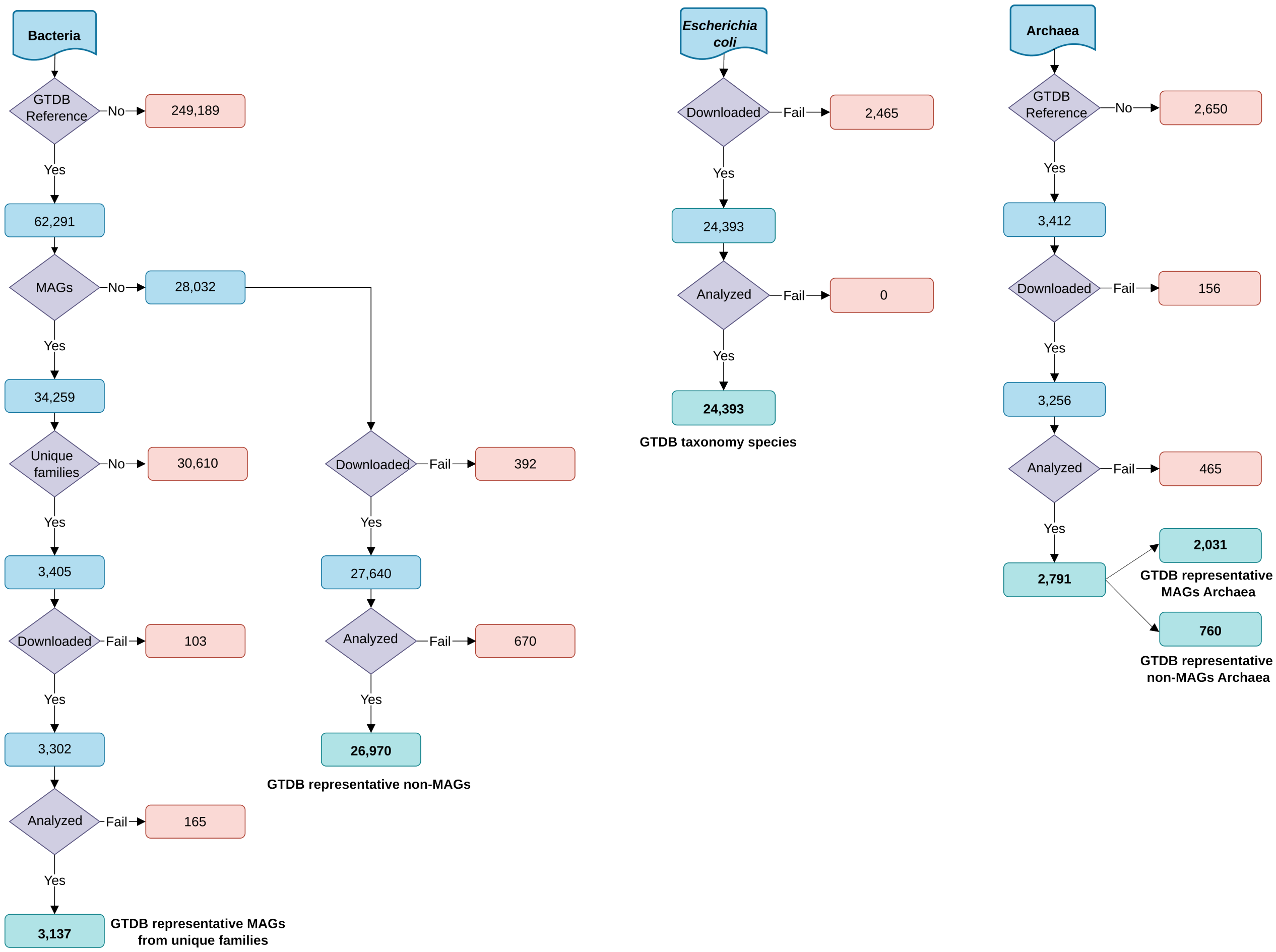

Fig. S1: Flowchart of the sample-selection process. The blue boxes at the top represent the groups investigated in the analysis. Purple rhombuses indicate decision points where samples may be excluded. Green boxes show the number of samples that pass each decision point, while red boxes show the number of samples that are rejected or fail at each stage.

2 Dataset composition

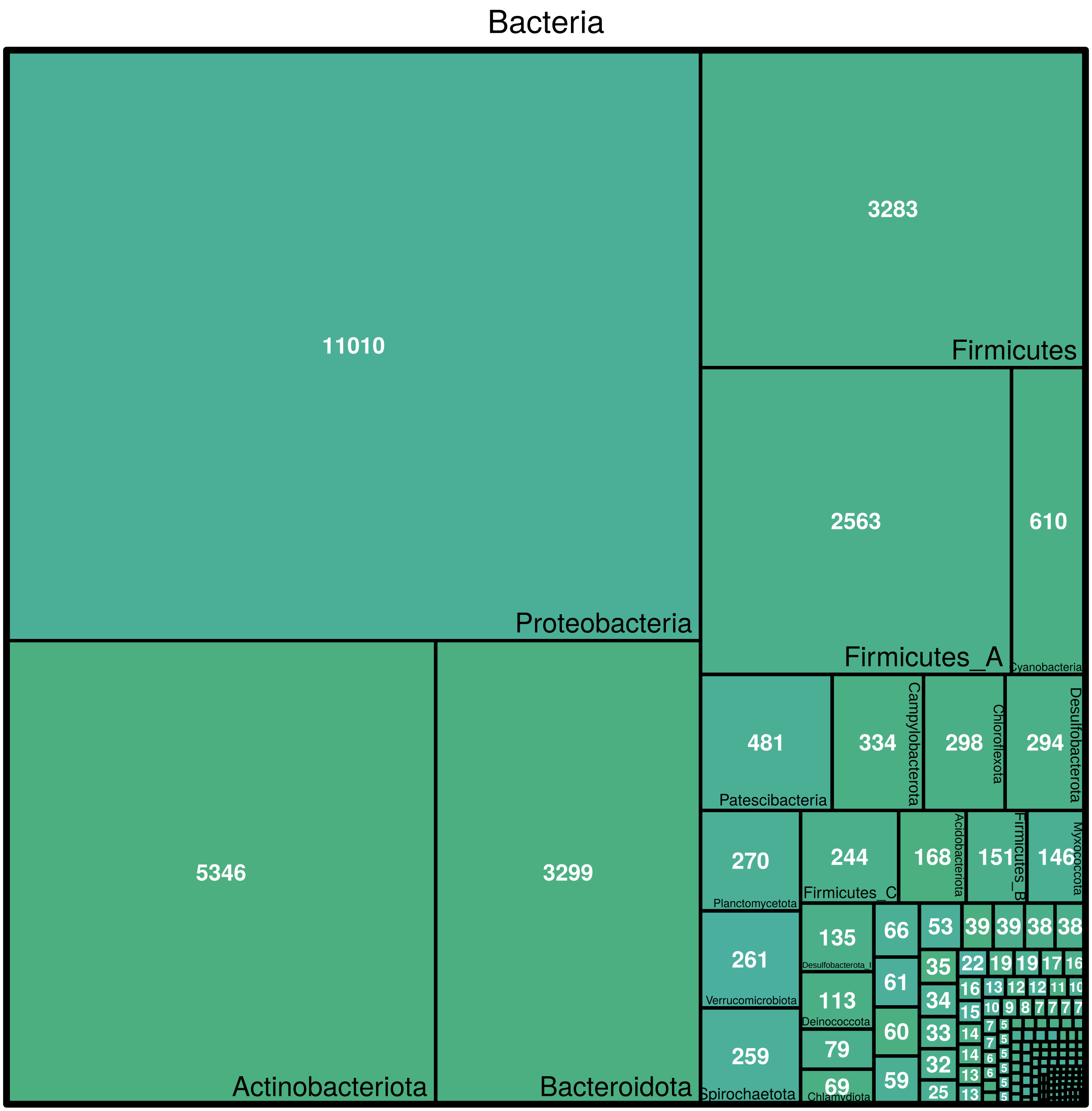

Fig. S2: The composition of the Bacteria kingdom, divided by phylum. The *E. coli* baseline annotation group is excluded from the visualization. Phylum nomenclature is based on GTDB (version 107). Only phyla with more than 100 representatives are labeled.

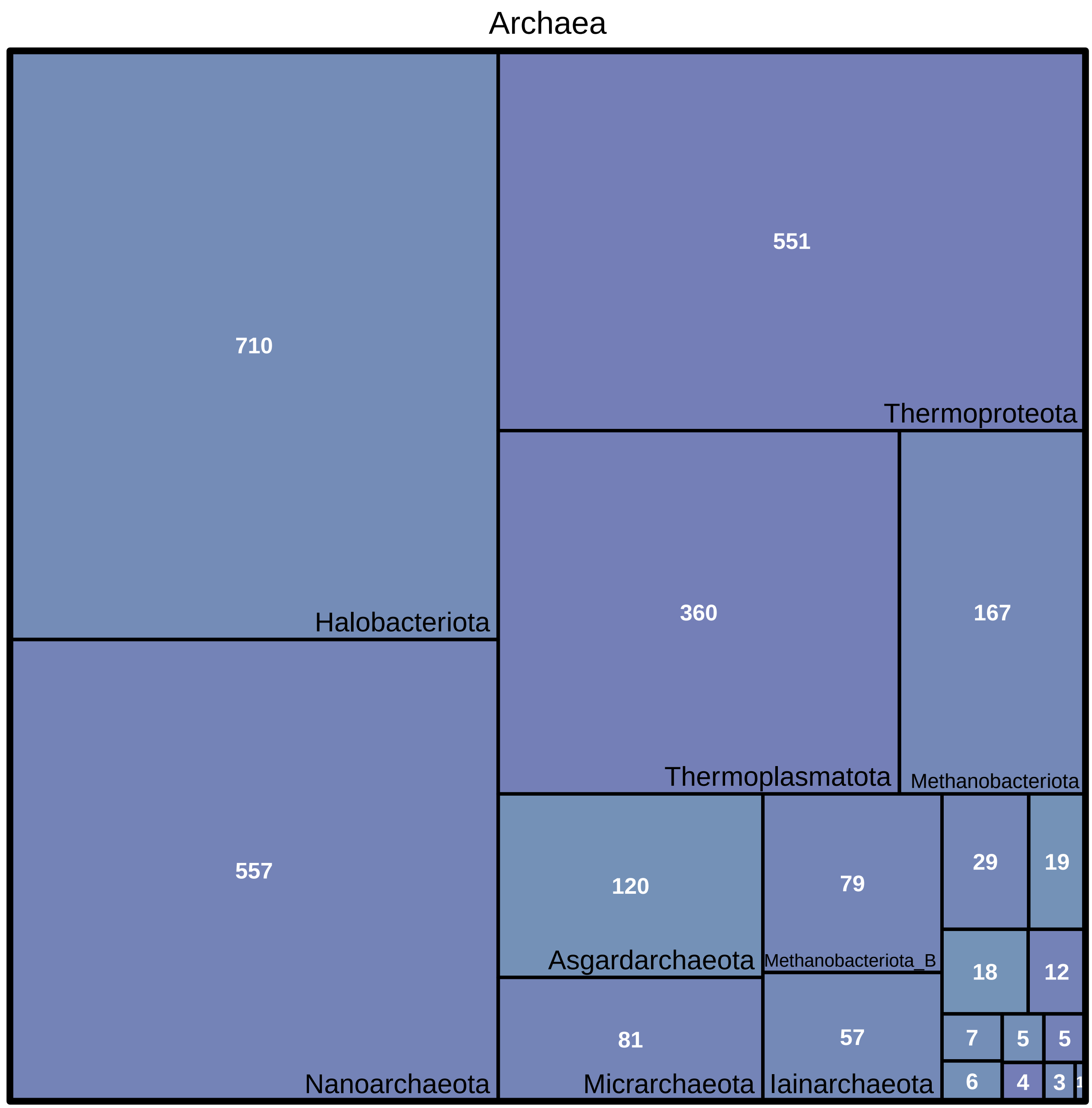

Fig. S3: The composition of the Archaea kingdom, divided by phylum. Phylum nomenclature is based on GTDB (version 107). Only phyla with more than 50 representatives are labeled.

3 Baseline annotation (*E. coli*)

3.1 Data description for *E. coli* strains

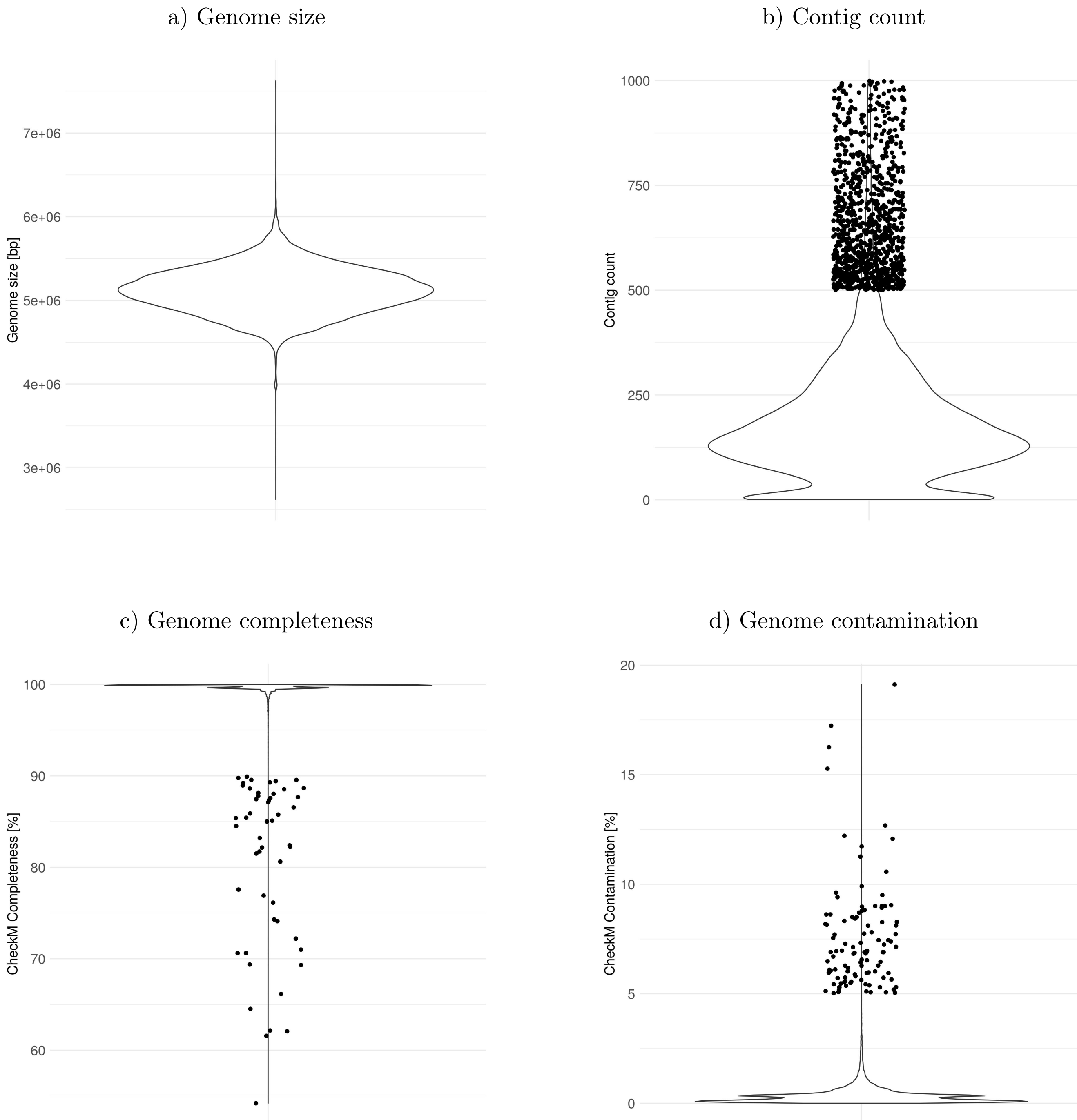

Fig. S4: Basic metrics characterizing the *Escherichia coli* subgroup within the analyzed samples. Each panel presents a distinct attribute reported in the GTDB dataset: (A) genome size derived from the genome_size variable, (B) contig count derived from the contig_count variable, (C) genome completeness derived from the GTDB checkm_completeness variable, and (D) genome contamination derived from the GTDB checkm_contamination variable.

3.2 Differences in assembly-gap annotation between tools

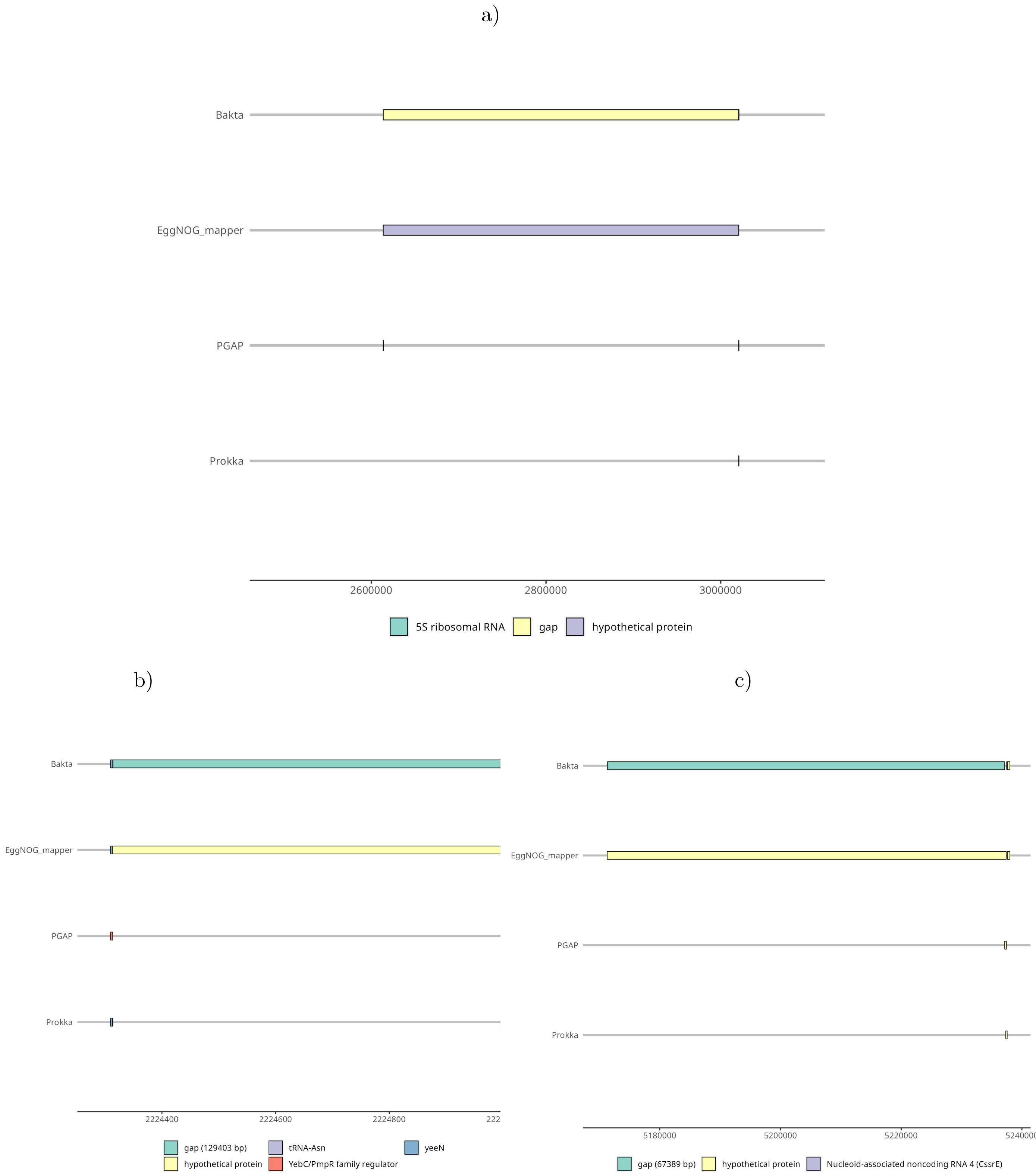

Fig. S5: Examples of unusually long hypothetical proteins identified by EggNOG-mapper in GCA_014216915.1. The genomic regions containing these anomalies are: (a) 2490000 to 3090000 bp, (b) 2224285 to 2355669 bp, and (c) 5170000 to 5239000 bp.

3.3 Total feature count

Table S1: Comparative results for total feature count and the one-way ANOVA with Tukey's HSD post hoc test. As an example, the Prokka-versus-Bakta comparison means that Prokka found, on average, 349.00117 fewer features than Bakta.

Comparison	Mean difference [N]	Lower endpoint of 95% CI	Upper endpoint of 95% CI	Adjusted P-value
Prokka vs Bakta	-349.00117	-357.97468	-340.02767	< 0.001
Prokka vs EggNOG-mapper	-20.84123	-29.81474	-11.86772	< 0.001
Prokka vs PGAP	-561.18322	-570.15673	-552.20971	< 0.001
EggNOG-mapper vs Bakta	-328.19206	-337.16781	-319.21632	< 0.001
PGAP vs Bakta	212.14931	203.17356	221.12505	< 0.001
PGAP vs EggNOG-mapper	540.34137	531.36562	549.31712	< 0.001

3.4 Undescribed feature count

Table S2: Comparative results for undescribed feature count and the one-way ANOVA with Tukey's HSD post hoc test. As an example, the Prokka-versus-Bakta comparison means that Prokka had, on average, 1101.9237 more undescribed features than Bakta.

Comparison	Mean difference [N]	Lower endpoint of 95% CI	Upper endpoint of 95% CI	Adjusted P-value
Prokka vs Bakta	1101.9237	1097.8702	1105.9773	< 0.001
Prokka vs EggNOG-mapper	706.1617	702.1082	710.2153	< 0.001
Prokka vs PGAP	398.1793	394.1258	402.2329	< 0.001
EggNOG-mapper vs Bakta	395.7409	391.5039	399.9779	< 0.001
PGAP vs Bakta	703.7365	699.4995	707.9735	< 0.001
PGAP vs EggNOG-mapper	307.9956	303.7586	312.2326	< 0.001

3.5 Total feature length

Table S3: Comparative results for total feature length and the one-way ANOVA with Tukey's HSD post hoc test. As an example, the Prokka-versus-Bakta comparison means that Prokka had, on average, features that were 4.5116172 aa longer than Bakta's.

Comparison	Mean difference [aa]	Lower endpoint of 95% CI	Upper endpoint of 95% CI	Adjusted P-value
Prokka vs Bakta	4.5116172	4.2865141	4.7367202	< 0.001
Prokka vs EggNOG-mapper	3.0744184	2.8493153	3.2995214	< 0.001
Prokka vs PGAP	3.9286927	3.7035897	4.1537958	< 0.001
EggNOG-mapper vs Bakta	1.4380230	1.2130425	1.6630036	< 0.001
PGAP vs Bakta	0.5838754	0.3588949	0.8088560	< 0.001
PGAP vs EggNOG-mapper	-0.8541476	-1.0791282	-0.6291671	< 0.001

3.6 Undescribed feature length

Table S4: Comparative results for undescribed feature length and the one-way ANOVA with Tukey's HSD post hoc test. As an example, the Prokka-versus-Bakta comparison means that Prokka had, on average, features that were 106.26033 aa longer than Bakta's.

Comparison	Mean difference [aa]	Lower endpoint of 95% CI	Upper endpoint of 95% CI	Adjusted P-value
Prokka vs Bakta	106.26033	105.18910	107.33157	< 0.001
Prokka vs EggNOG-mapper	-22.36681	-23.43804	-21.29557	0.9835263
Prokka vs PGAP	92.02810	90.95686	93.09933	< 0.001
EggNOG-mapper vs Bakta	128.622926	127.5501071	129.6957460	< 0.001
PGAP vs Bakta	14.235710	13.1628907	15.3085290	< 0.001
PGAP vs EggNOG-mapper	-114.387216	-115.4600358	-113.3143970	< 0.001

3.7 Total annotated coding space

Table S5: Comparative results for total annotated coding space and the one-way ANOVA with Tukey's HSD post hoc test. As an example, the Prokka-versus-Bakta comparison means that Prokka had, on average, a total coding space that was 39921.00 bp smaller than Bakta's.

Comparison	Mean difference [bp]	Lower endpoint of 95% CI	Upper endpoint of 95% CI	Adjusted P-value
Prokka vs Bakta	-39921.00	-45711.101	-34130.905	< 0.001
Prokka vs EggNOG-mapper	-41519.55	-47309.651	-35729.454	< 0.001
Prokka vs PGAP	-251601.62	-257391.723	-245811.527	< 0.001
EggNOG-mapper vs Bakta	1582.169	-4209.256	7373.595	0.896
PGAP vs Bakta	211660.417	205868.992	217451.843	< 0.001
PGAP vs EggNOG-mapper	210078.248	204286.822	215869.674	< 0.001

3.8 Described coding space

Table S6: Comparative results for described coding space and the one-way ANOVA with Tukey's HSD post hoc test. As an example, the Prokka-versus-Bakta comparison means that Prokka had, on average, a described coding space that was 770335.71 bp smaller than Bakta's.

Comparison	Mean difference [bp]	Lower endpoint of 95% CI	Upper endpoint of 95% CI	Adjusted P-value
Prokka vs Bakta	-770335.71	-774780.14	-765891.27	< 0.001
Prokka vs EggNOG-mapper	-633092.87	-637537.30	-628648.43	< 0.001
Prokka vs PGAP	-716555.28	-720999.71	-712110.84	< 0.001
EggNOG-mapper vs Bakta	-137250.13	-141676.91	-132823.34	< 0.001
PGAP vs Bakta	-53789.66	-58216.44	-49362.87	< 0.001
PGAP vs EggNOG-mapper	83460.47	79033.68	87887.26	< 0.001

3.9 Undescribed coding space

Table S7: Comparative results for undescribed coding space and the one-way ANOVA with Tukey's HSD post hoc test. As an example, the Prokka-versus-Bakta comparison means that Prokka had, on average, an undescribed coding space that was 729400.22 bp larger than Bakta's.

Comparison	Mean difference [bp]	Lower endpoint of 95% CI	Upper endpoint of 95% CI	Adjusted P-value
Prokka vs Bakta	729400.22	727533.69	731266.74	< 0.001
Prokka vs EggNOG-mapper	590558.83	588692.30	592425.35	< 0.001
Prokka vs PGAP	631547.63	629681.11	633414.15	< 0.001
EggNOG-mapper vs Bakta	138832.30	136886.93	140777.67	< 0.001
PGAP vs Bakta	97849.72	95904.35	99795.08	< 0.001
PGAP vs EggNOG-mapper	-40982.58	-42927.95	-39037.21	< 0.001

3.10 Impact on rRNA and tRNA discoverability in *E. coli*

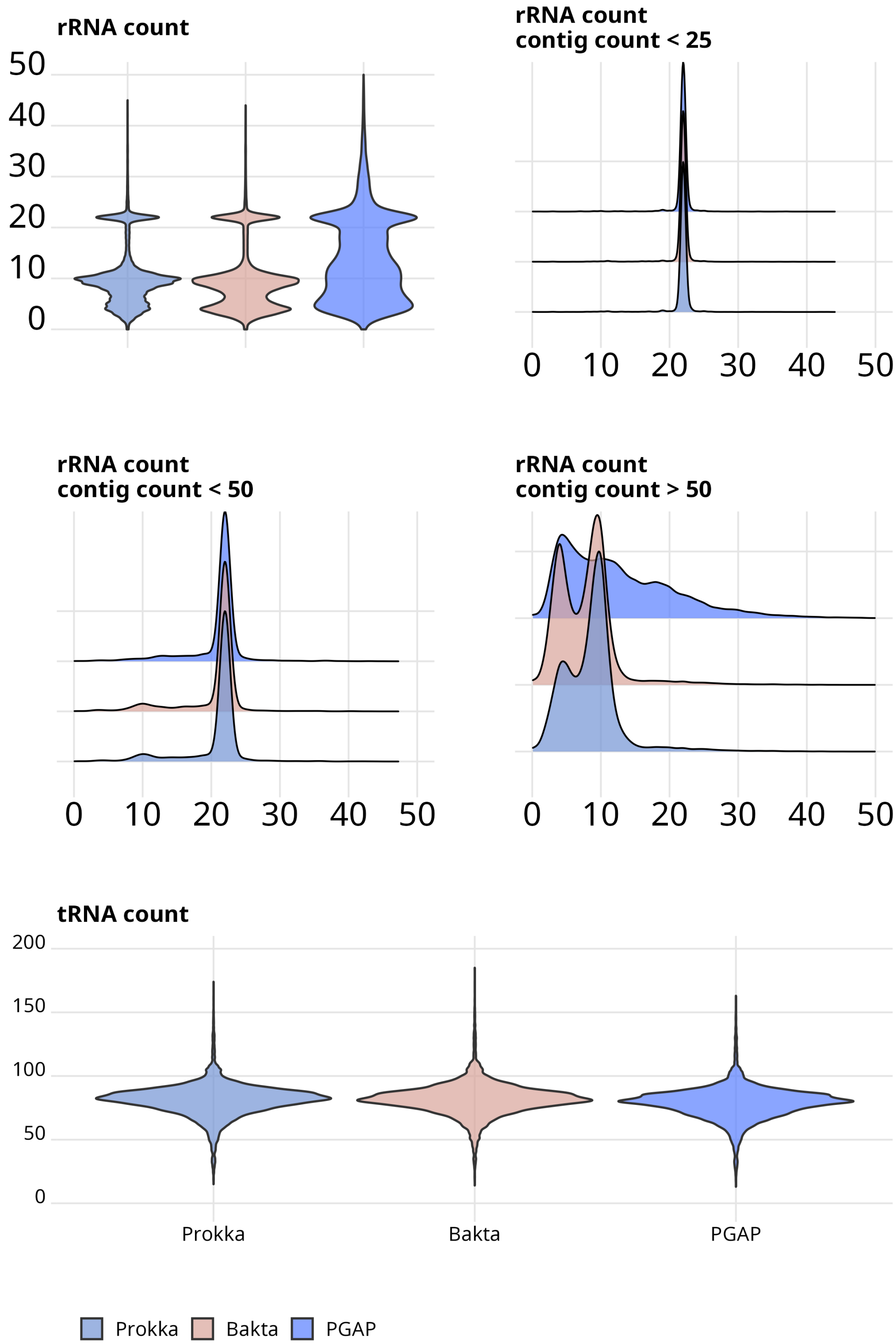

Fig. S6: Analysis of contig counts and their impact on rRNA and tRNA discoverability in *Escherichia coli*. (a) rRNA identification across contig-count bins, with a ridge plot showing the rRNA distribution in three groups ($<$25, $<$50, and $>$50 contigs), along with the distribution of tRNA counts.

4 Quality analysis of GO terms in *Escherichia coli* K-12 substr. MDS42

Table S8: Summary statistics for the hierarchical levels of GO terms obtained from the GOxploreR R package for *Escherichia coli* K-12 substr. MDS42 (GCA_000350185.1) across annotation tools. Mean, median, standard deviation (SD), minimum, and maximum levels are shown for each tool, reported separately for the BP (biological process), MF (molecular function), and CC (cellular component) ontologies. N represents the total number of annotated GO terms; higher levels indicate more specific annotations.

Tool	Ontology	Mean level	Median level	SD	Minimum level	Maximum level	Total GO count
Bakta	BP	6.82	7	2.40	1	15	5107
Bakta	CC	3.45	3	1.23	1	10	3180
Bakta	MF	4.97	5	1.83	1	10	6330
EggNOG-mapper	BP	4.48	4	2.50	0	14	53699
EggNOG-mapper	CC	2.30	2	1.54	0	10	11579
EggNOG-mapper	MF	3.18	3	1.95	0	10	26108
PGAP	BP	7.01	7	2.38	0	14	1542
PGAP	CC	4.40	3	2.32	0	9	362
PGAP	MF	4.76	5	1.88	0	9	2003

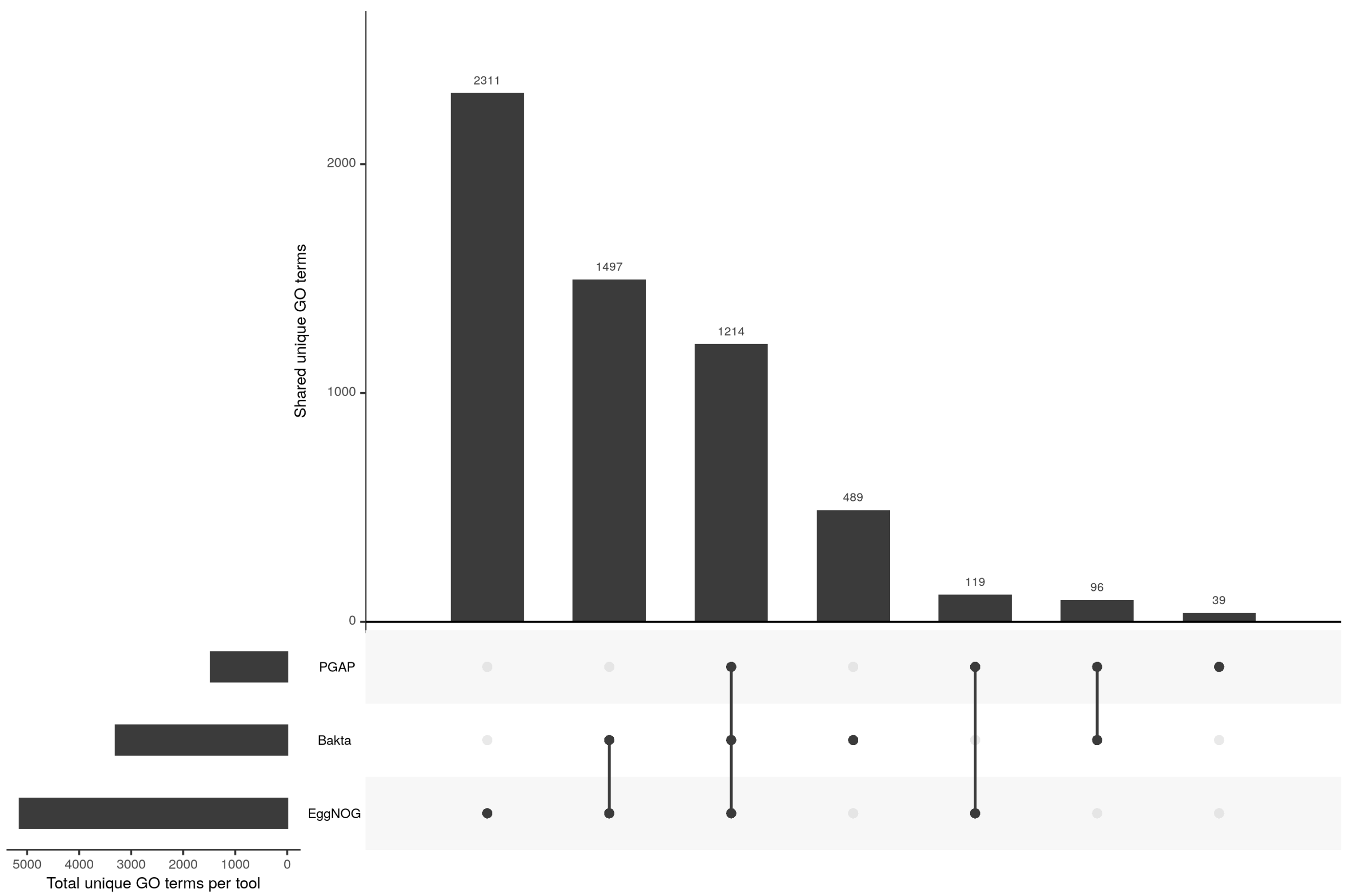

Fig. S7: An UpSet plot visualizes the intersection of unique GO term annotations among Bakta, PGAP, and EggNOG-mapper for *Escherichia coli* K-12 substr. MDS42 (GCA_000350185.1). Horizontal bars (left) show the total number of unique GO terms per tool; vertical bars indicate intersection sizes, with connected dots denoting shared terms.

5 The Bacteria and Archaea kingdoms

5.1 Data description for the Bacteria kingdom

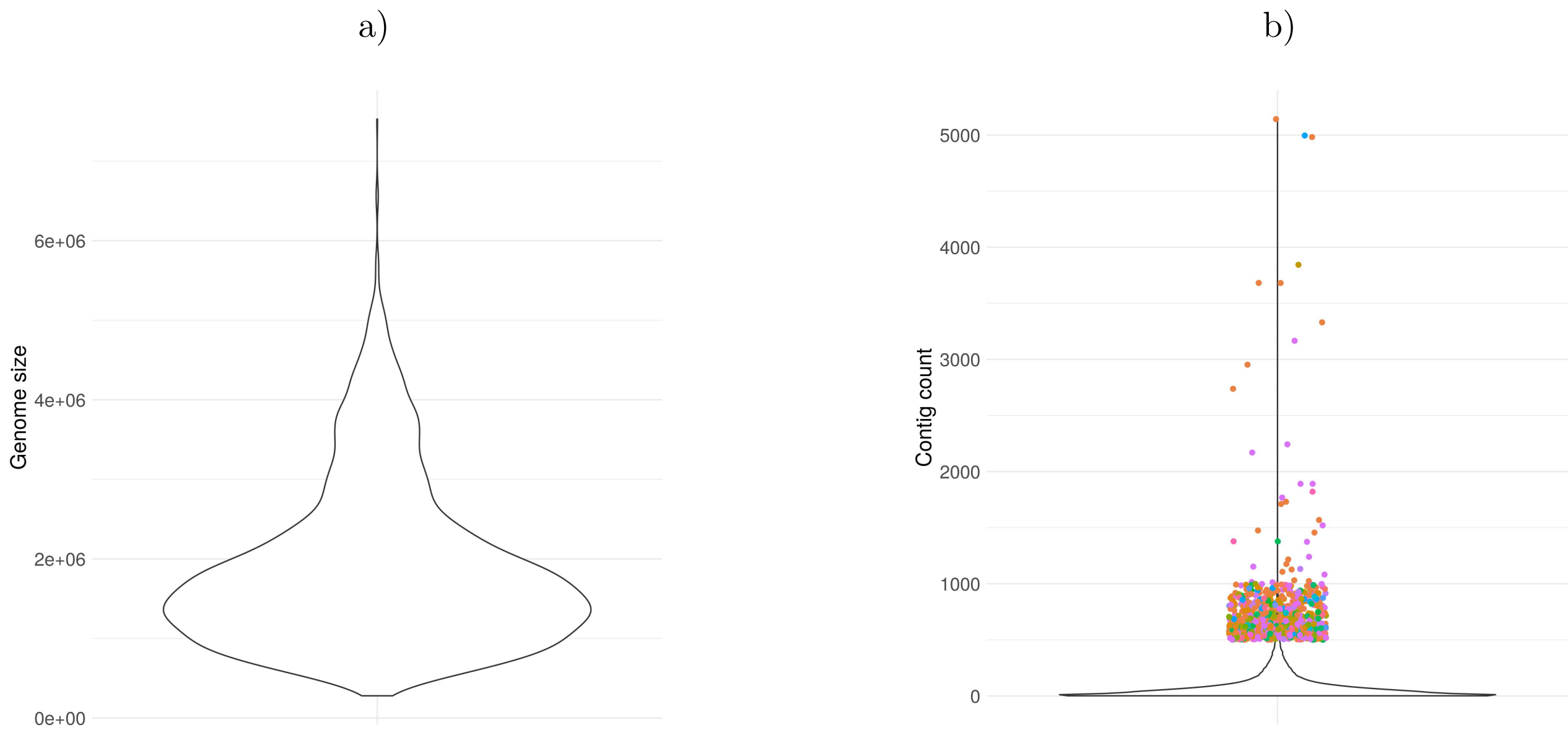

Fig. S8: Basic metrics characterizing the Bacteria kingdom within the analyzed samples. Each panel presents a distinct attribute: (A) genome size derived from the genome_size variable and (B) contig count derived from the contig_count variable. Major outliers in the respective categories are shown as dots, color-coded according to their phyla as recorded in the gtdb_taxonomy variable.

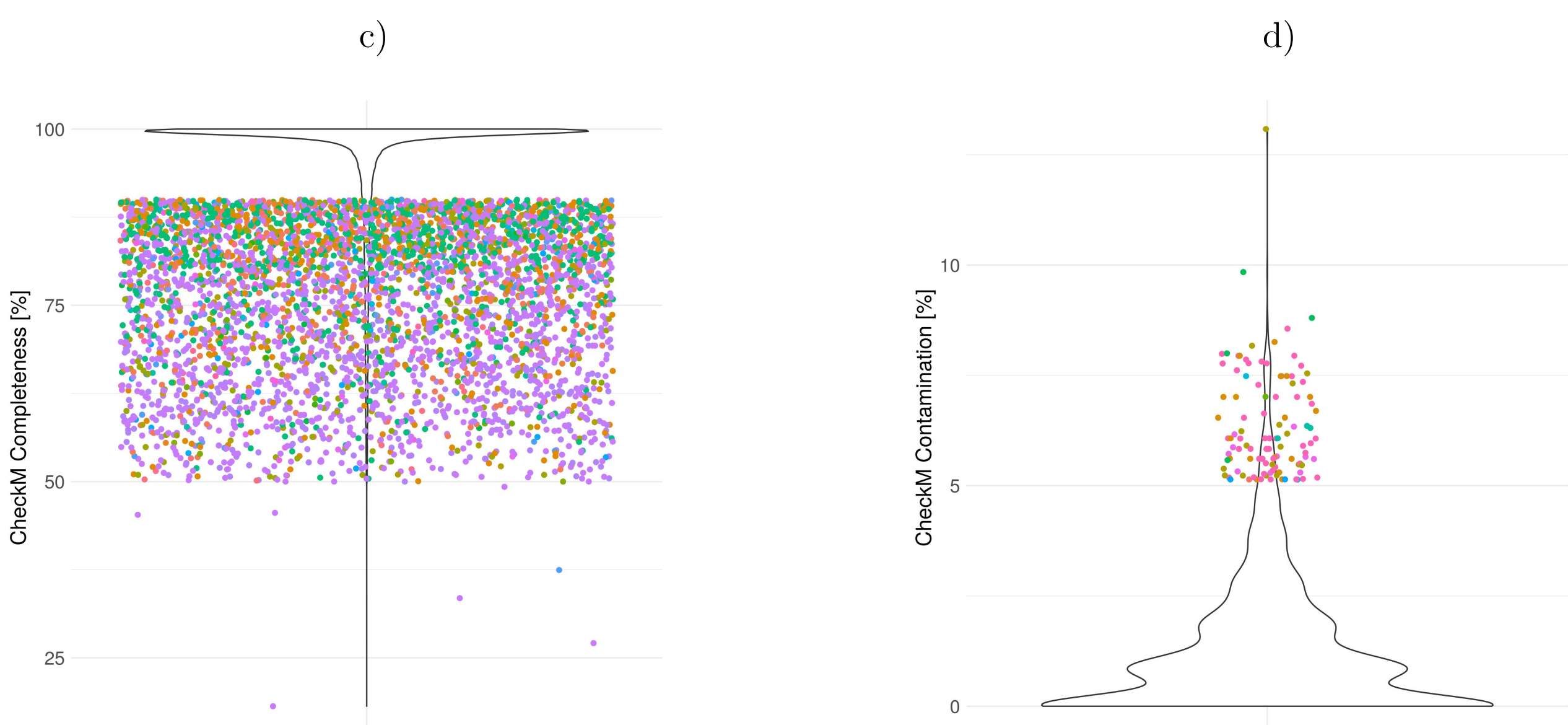

Fig. S9: Basic metrics characterizing the Bacteria kingdom within the analyzed samples. Each panel presents a distinct attribute: (C) genome completeness derived from the GTDB checkm_completeness variable and (D) genome contamination derived from the GTDB checkm_contamination variable. Major outliers in the respective categories are shown as dots, color-coded according to their phyla as recorded in the gtdb_taxonomy variable.

5.2 Data description for the Archaea kingdom

a) b)

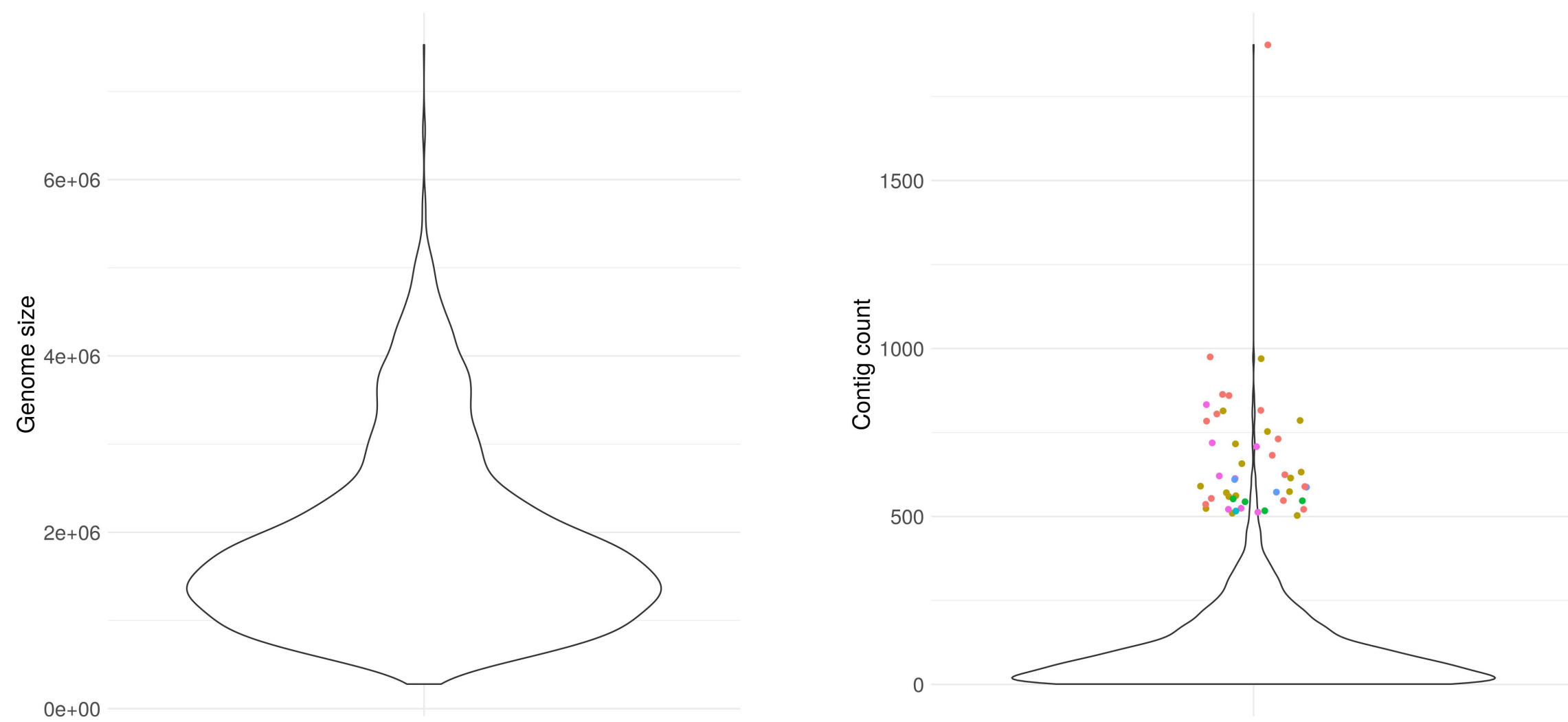

Fig. S10: Basic metrics characterizing the Archaea subgroup within the analyzed samples. Each panel presents a distinct attribute: (A) genome size derived from the genome_size variable and (B) contig count derived from the contig_count variable. Major outliers in the respective categories are shown as dots, color-coded according to their phyla as recorded in the gtdb_taxonomy variable.

c) d)

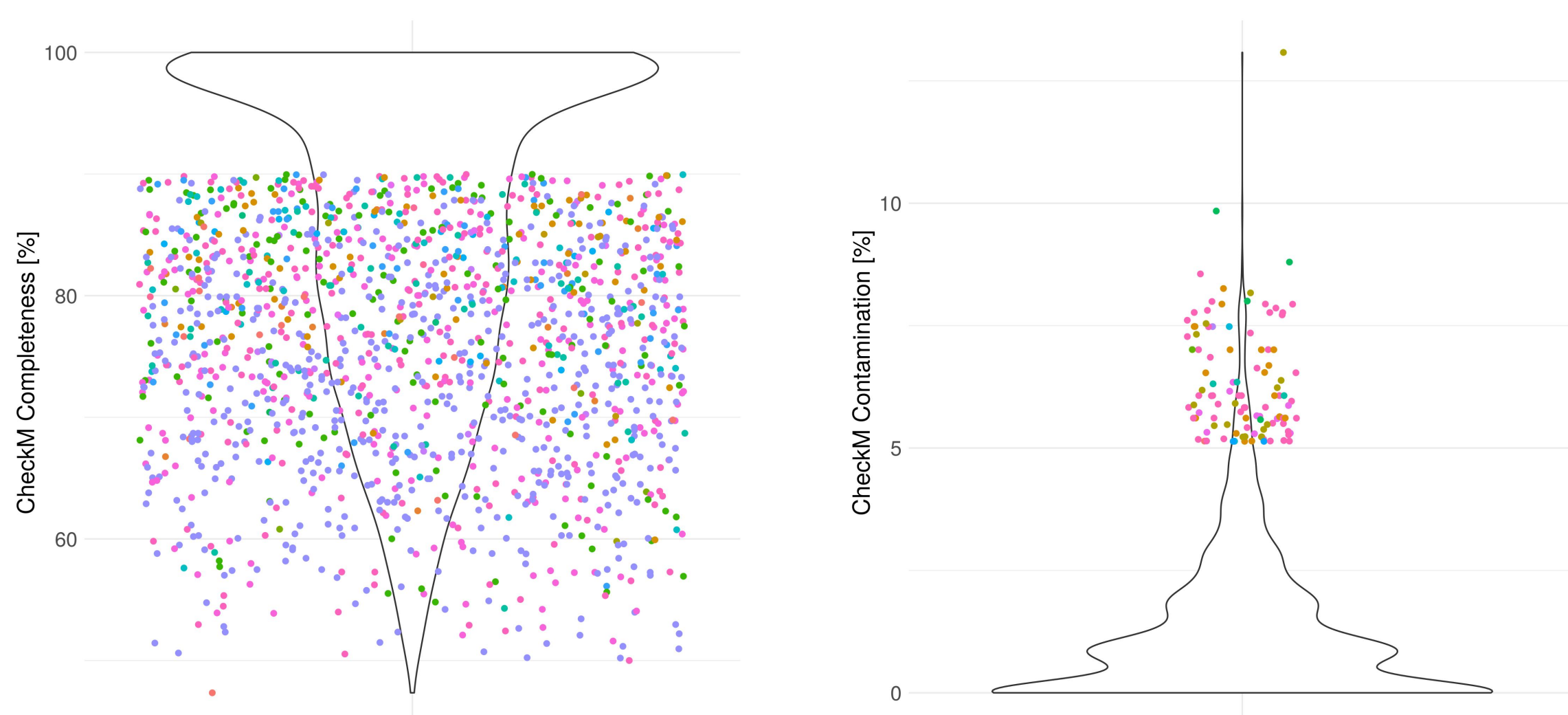

Fig. S11: Basic metrics characterizing the Archaea subgroup within the analyzed samples. Each panel presents a distinct attribute: (C) genome completeness derived from the GTDB checkm_completeness variable and (D) genome contamination derived from the GTDB checkm_contamination variable. Major outliers in the respective categories are shown as dots, color-coded according to their phyla as recorded in the gtdb_taxonomy variable.

5.3 Total coding density in the Bacteria kingdom

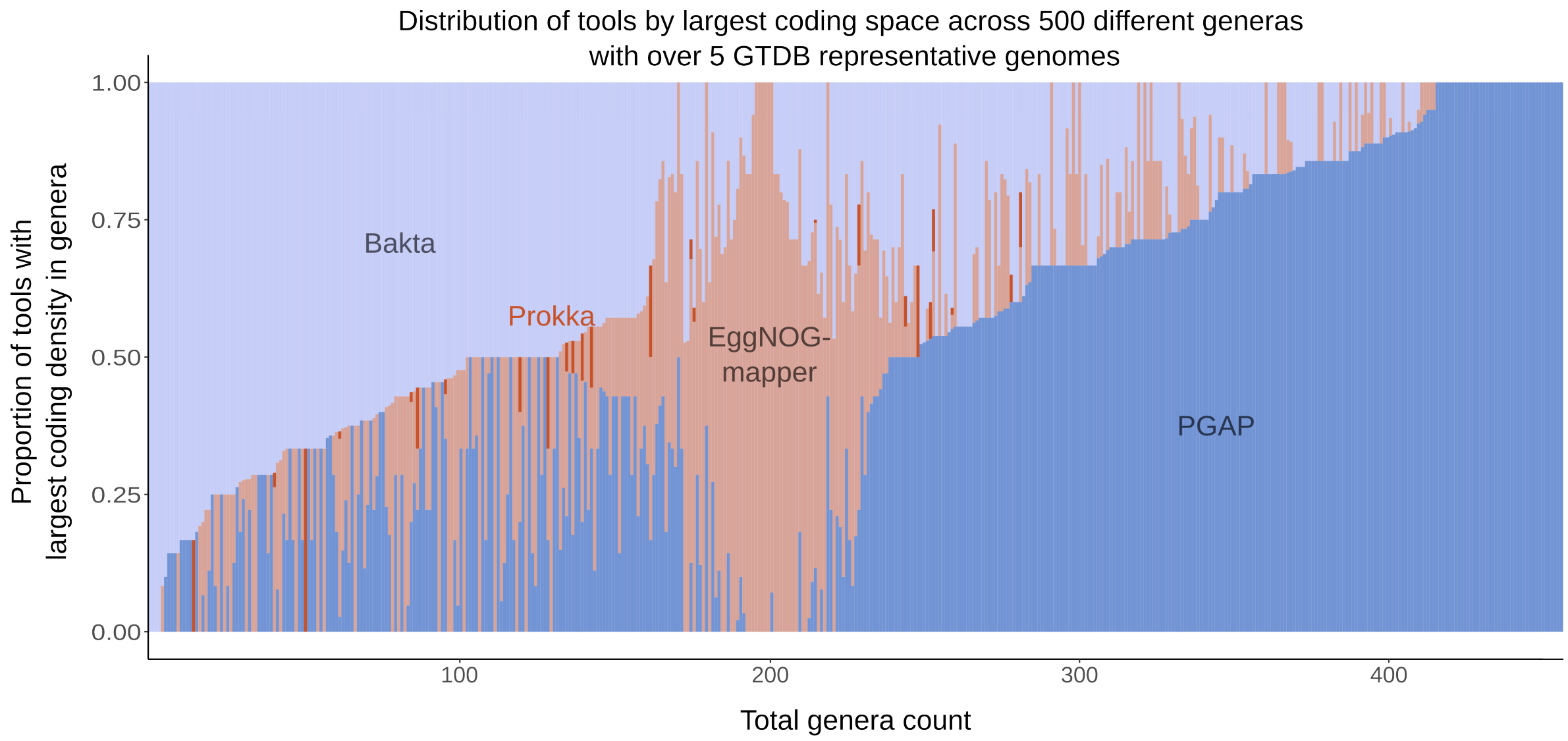

Fig. S12: Comparison of tool performance in achieving the highest total coding density for species grouped by genus. The bar charts show the number of times a tool achieved the highest total coding density. The difference in performance between the best- and second-best-performing tools was not considered in this approach.

5.4 Feature length comparison in the Bacteria kingdom

a)

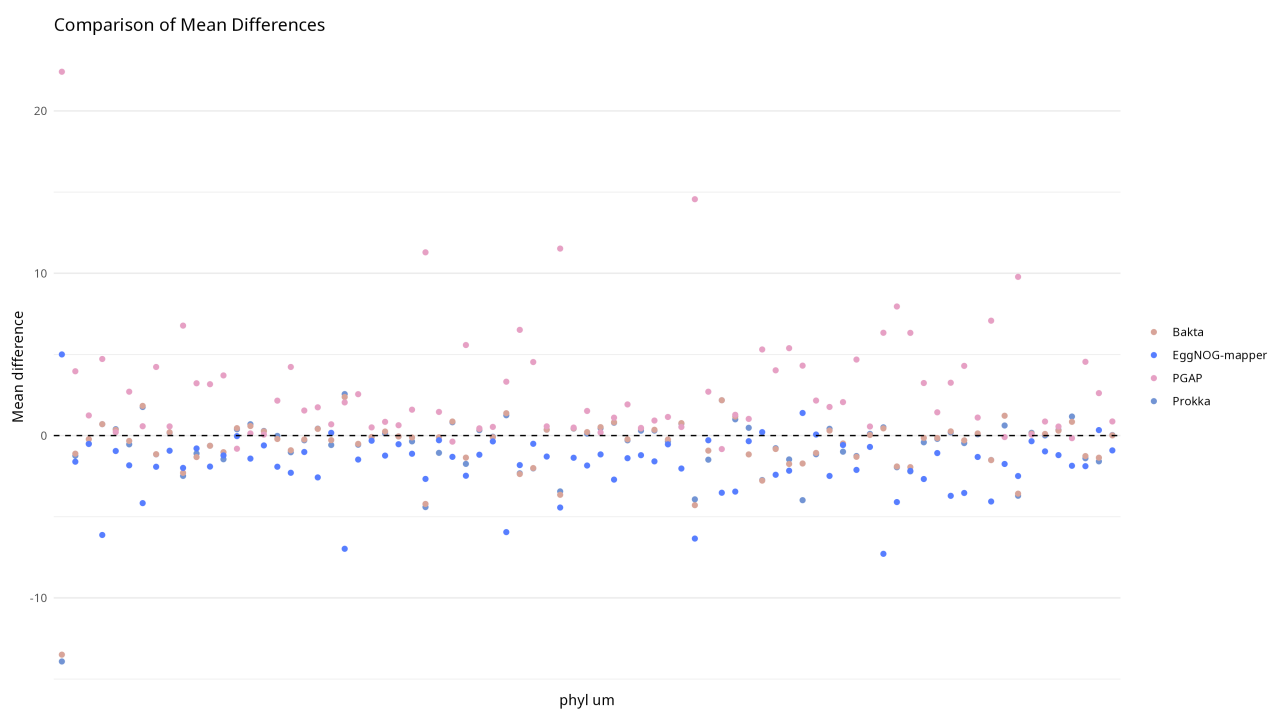

b)

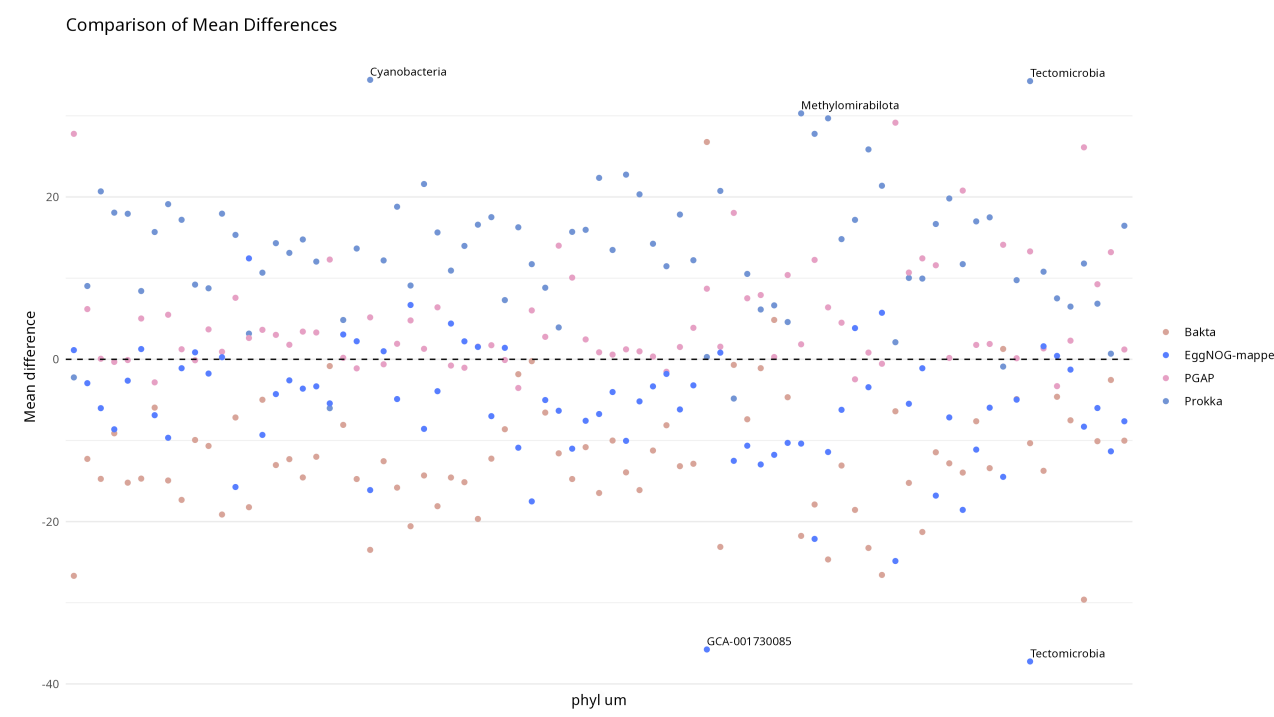

c)

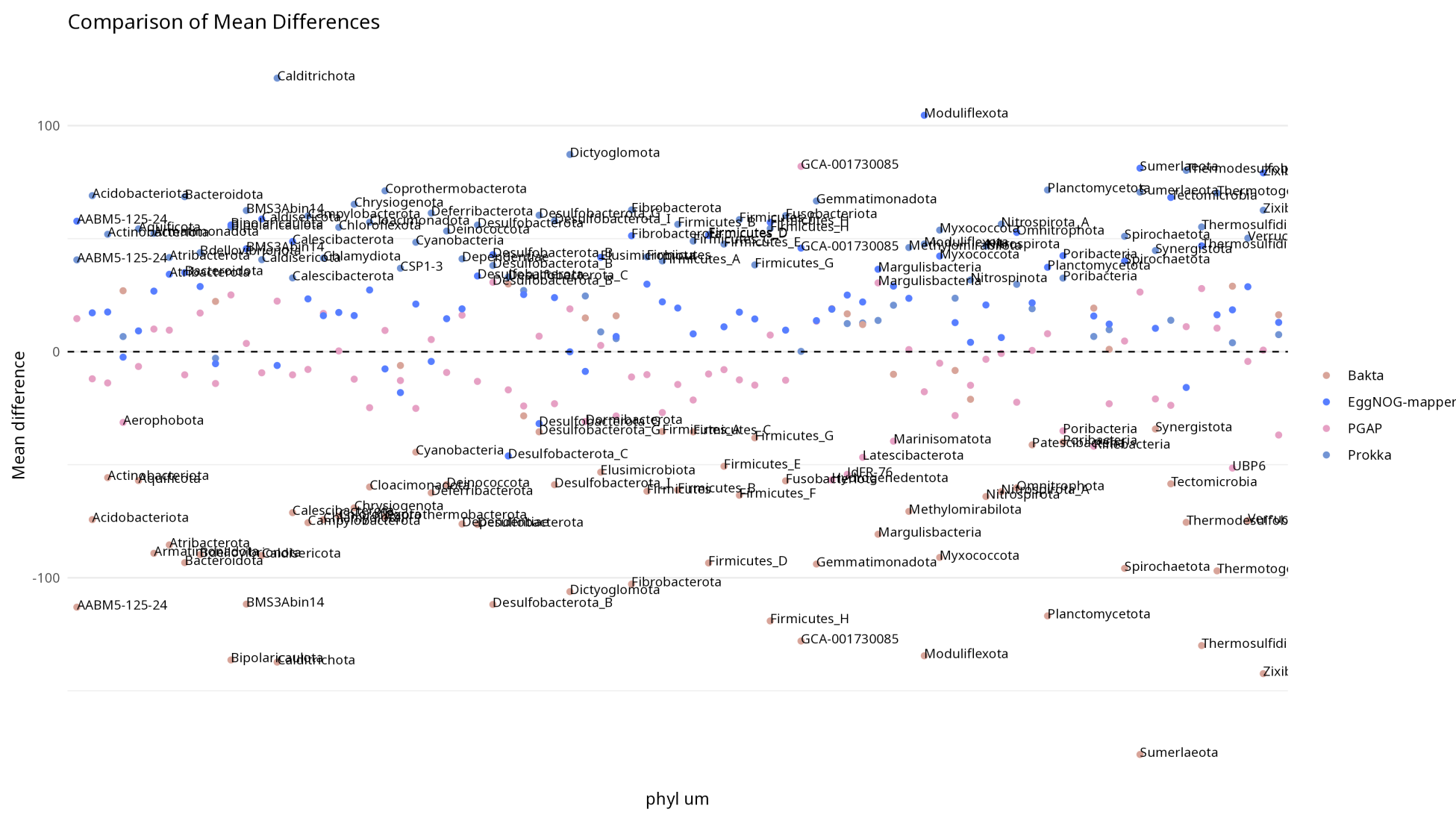

Fig. S13: Comparison of mean differences in feature lengths for Prokka, Bakta, EggNOG-mapper, and PGAP across various phyla. Dashed horizontal lines at y = 0 indicate no mean difference. Subplot (a) shows differences in the mean lengths of all features (including hypothetical and fully annotated features), with phylum names annotated when the absolute difference exceeds 30. Subplot (b) displays differences in the mean lengths of described features, with annotations for absolute differences greater than 30. Subplot (c) illustrates differences in the mean lengths of undescribed features, with annotations for absolute differences greater than 100.

5.5 Feature length comparison in the Archaea kingdom

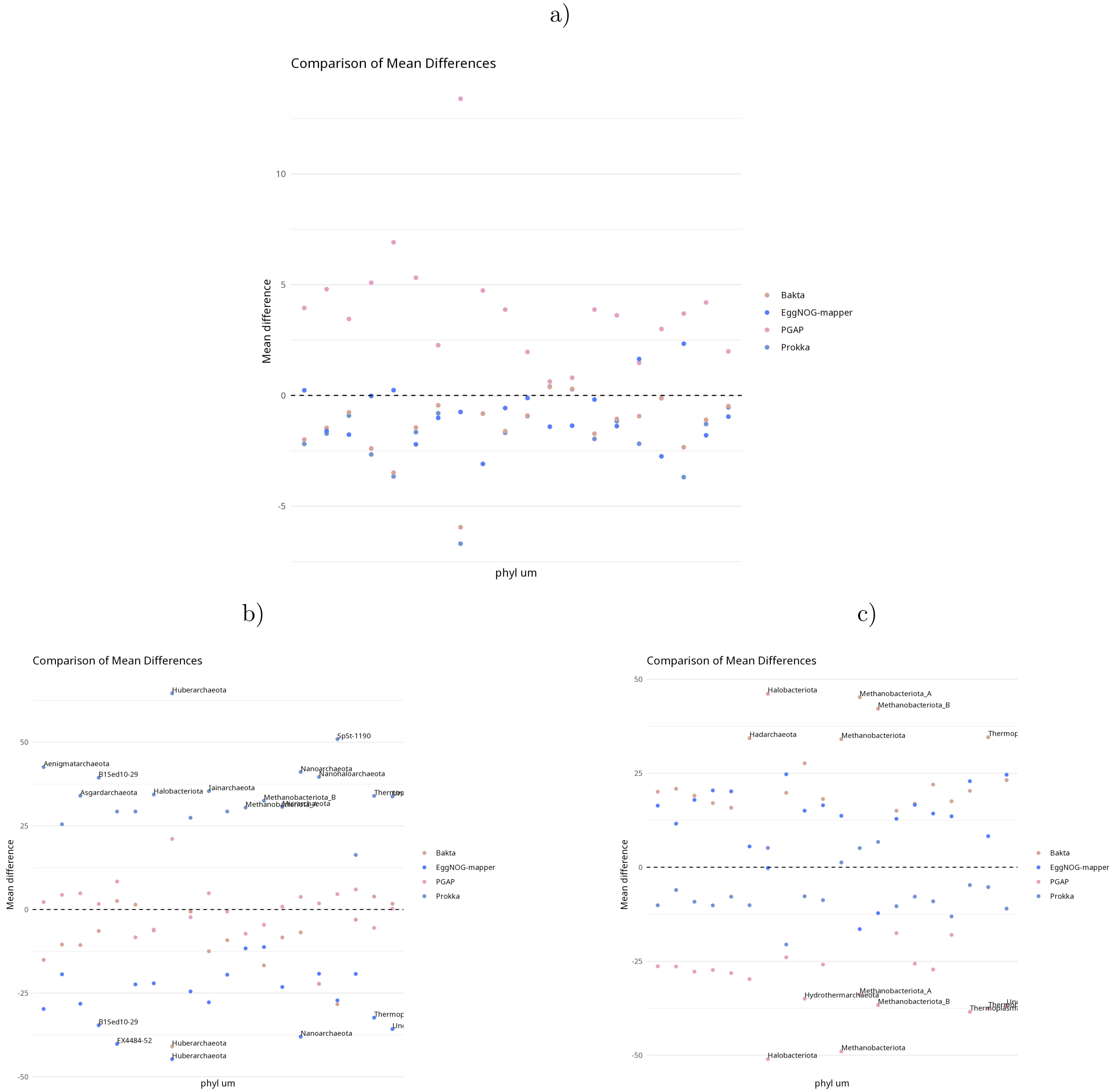

Fig. S14: Comparison of mean differences in feature lengths among tools across phyla. Dashed horizontal lines at y = 0 denote no difference in means. (a) shows the difference in total mean length (including both hypothetical and fully annotated features); phylum names are annotated when the absolute difference from the mean across all tools exceeds 30. (b) shows the difference in described mean length, with phylum names annotated when the absolute difference exceeds 30. (c) shows the difference in undescribed mean length, with phylum names annotated when the absolute difference exceeds 100.

5.6 rRNA comparison for the Archaea kingdom

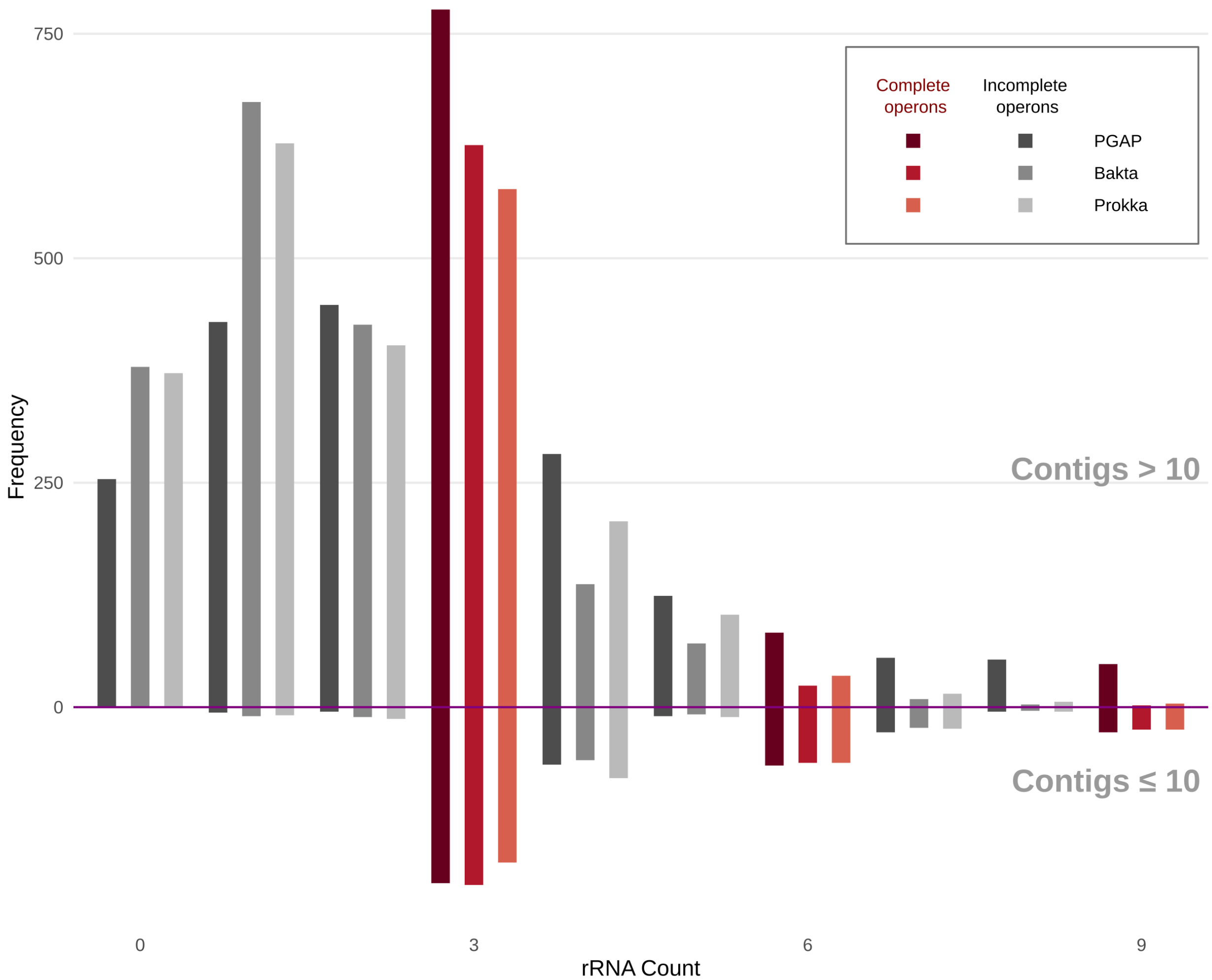

Fig. S15: Distribution of rRNA counts in complete and incomplete operons for each tool in the Archaea kingdom. The upper panel displays the more fragmented genomes, characterized by more than 10 contigs. In contrast, the bottom panel shows the less fragmented samples, with fewer than 10 contigs. Complete operons, containing a multiple of three rRNA genes, are highlighted in red, while incomplete operons are displayed in gray.

5.7 Analysis of rRNA prediction variance in the Bacteria kingdom

Table S9: Top 20 samples with the highest rRNA counts detected by Prokka

ID	Prokka rRNA	Bakta rRNA	PGAP rRNA	Contig count
GCA_003674045.1	134	65	143	277
GCA_002162355.1	108	108	108	1
GCA_001623875.1	86	82	84	25
GCA_002243515.1	80	80	80	1
GCA_900176885.1	67	65	64	41
GCA_900291985.1	59	55	54	16
GCA_004006295.1	54	54	54	2
GCA_008931805.1	53	53	53	2
GCA_002019645.1	52	49	48	8
GCA_002355375.1	51	51	51	1
GCA_013248975.1	51	51	51	3
GCA_900460535.1	50	49	50	5
GCA_900465055.1	50	50	50	1
GCA_000196255.1	49	49	49	3
GCA_002019605.1	49	49	49	17
GCA_900002575.1	49	48	49	1
GCA_002109385.1	47	47	47	1
GCA_016811915.1	47	47	47	1
GCA_900199725.1	47	46	40	69
GCA_014879975.1	46	46	46	2

Table S10: Top 20 samples with the highest rRNA counts detected by Bakta

ID	Prokka rRNA	Bakta rRNA	PGAP rRNA	Contig count
GCA_002162355.1	108	108	108	1
GCA_001623875.1	86	82	84	25
GCA_002243515.1	80	80	80	1
GCA_000686145.1	66	66	64	11
GCA_003674045.1	134	65	143	227
GCA_900176885.1	67	65	64	41
GCA_018219455.1	62	62	62	1
GCA_900177675.1	60	60	60	2
GCA_003667825.1	62	57	57	1
GCA_018219155.1	56	56	56	3
GCA_900291985.1	59	55	54	16
GCA_004006295.1	54	54	54	2
GCA_008931805.1	53	53	53	2
GCA_002355375.1	51	51	51	1
GCA_013248975.1	51	51	51	3
GCA_900465055.1	50	50	50	1
GCA_000196255.1	49	49	49	3
GCA_002019605.1	49	49	49	17
GCA_002019645.1	52	49	48	8
GCA_018223745.1	49	49	49	2

Table S11: Top 20 samples with the highest rRNA counts detected by PGAP

ID	Prokka rRNA	Bakta rRNA	PGAP rRNA	Contig count
GCA_000415505.1	22	17	227	1731
GCA_003674045.1	134	65	143	227
GCA_002162355.1	108	108	108	1
GCA_013359945.1	19	18	101	221
GCA_009720735.1	33	28	93	151
GCA_018403285.1	27	27	91	132
GCA_014502795.1	19	18	90	218
GCA_016587775.1	15	10	90	730
GCA_001623875.1	86	82	84	25
GCA_013359905.1	26	24	82	122
GCA_000986785.1	35	31	80	104
GCA_002243515.1	80	80	80	1
GCA_013359935.1	18	18	78	206
GCA_001635455.1	34	31	77	614
GCA_002257705.1	29	21	75	165
GCA_003148565.1	26	26	71	71
GCA_018332455.1	19	19	69	143
GCA_001659985.1	20	20	68	46
GCA_019219025.1	27	27	68	185
GCA_002897295.1	15	19	67	197

5.8 Capability of PGAP to annotate rRNA features in genomes with high contig counts

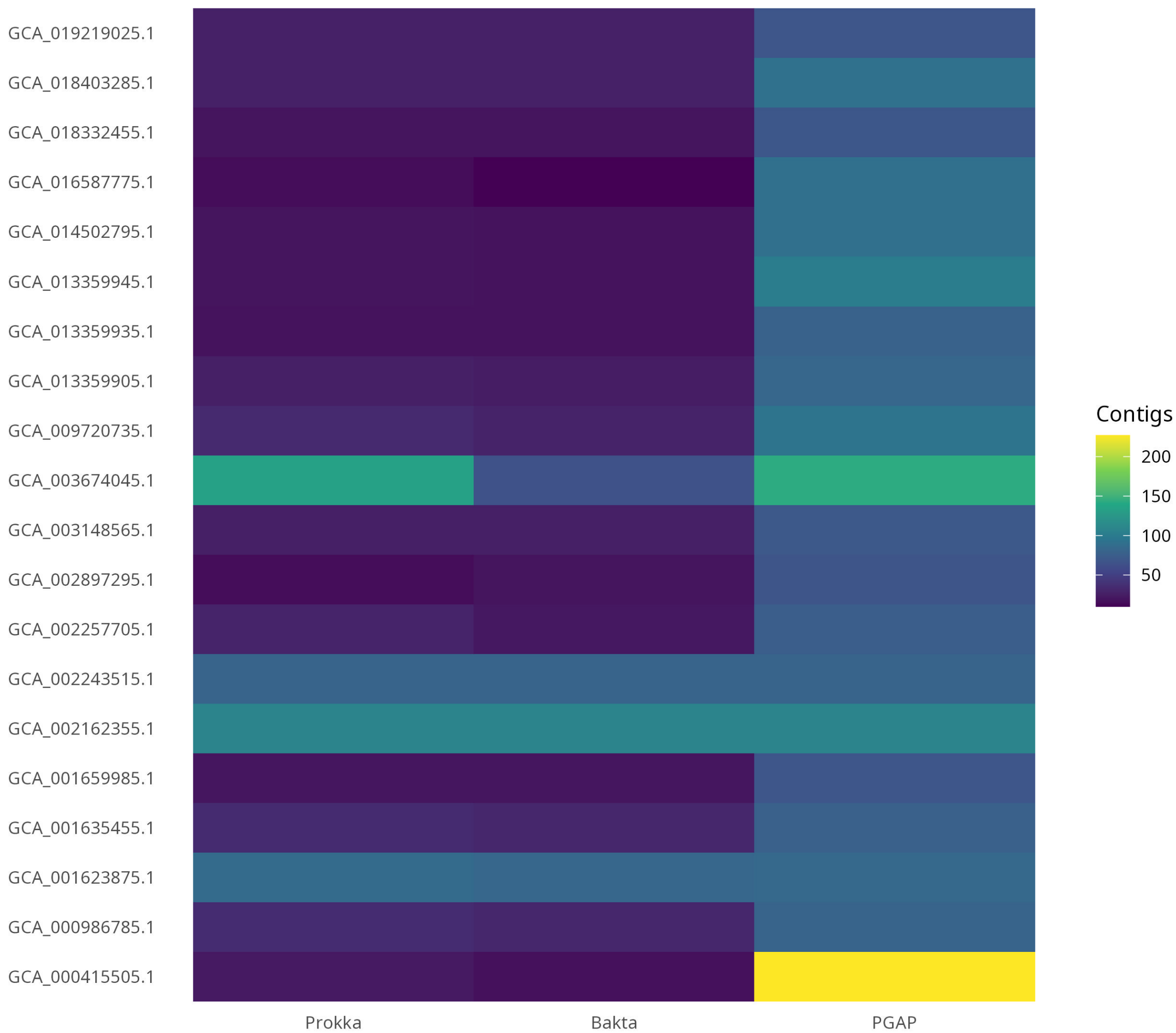

Fig. S16: Heatmap of the abundance of unique contigs with rRNA features in the 20 samples with the highest rRNA counts. Color intensity within each cell represents the relative abundance of unique contigs identified as containing rRNA sequences.

6 Metagenome-assembled genomes (MAGs)

6.1 Data description

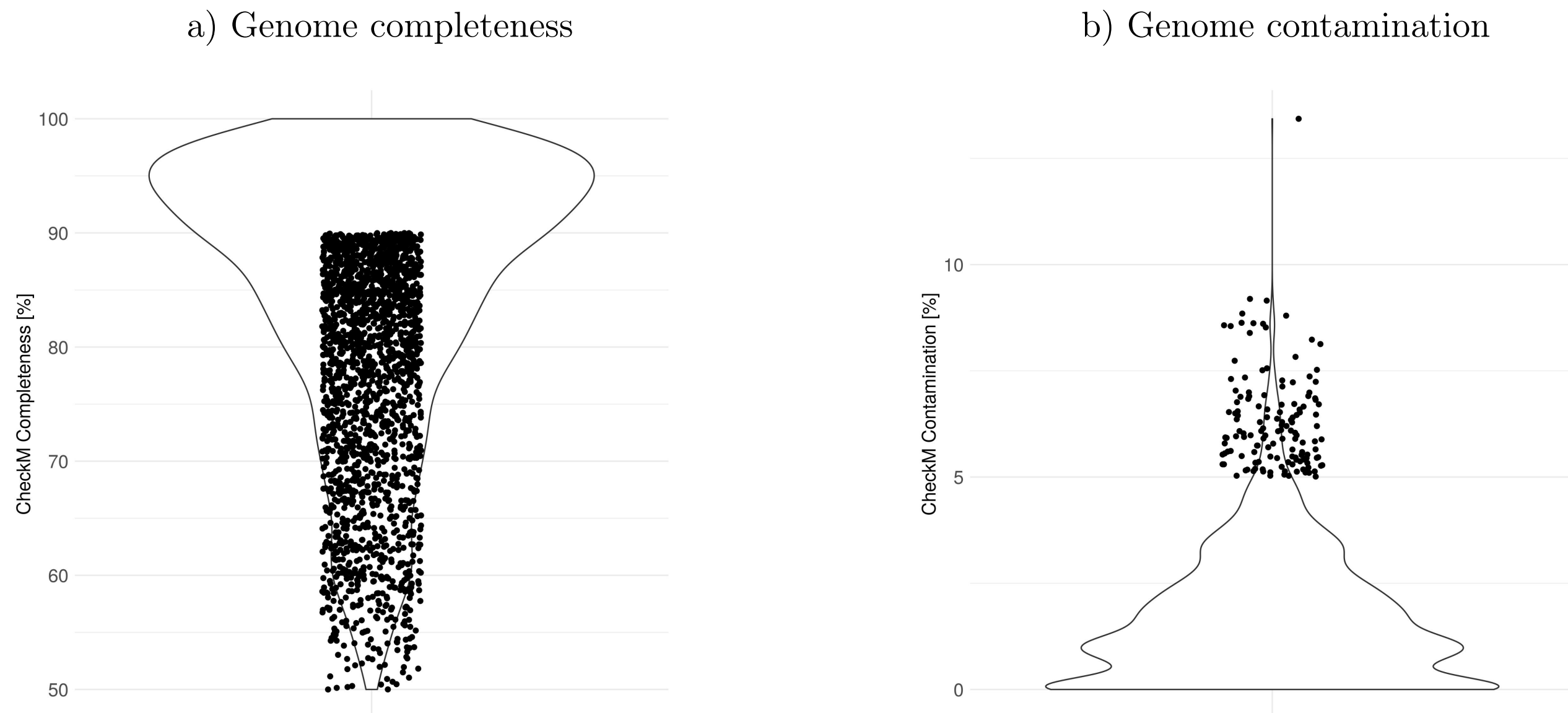

Fig. S17: Basic metrics characterizing the MAG subgroup within the analyzed samples. Each panel presents a distinct attribute reported in the GTDB/NCBI datasets: (a) genome completeness derived from the GTDB checkm_completeness variable and (b) genome contamination derived from the GTDB checkm_contamination variable.

6.2 Coding-length comparison

a) Length of undescribed proteins in the Bacteria kingdom

b) Length of all proteins in the Bacteria kingdom

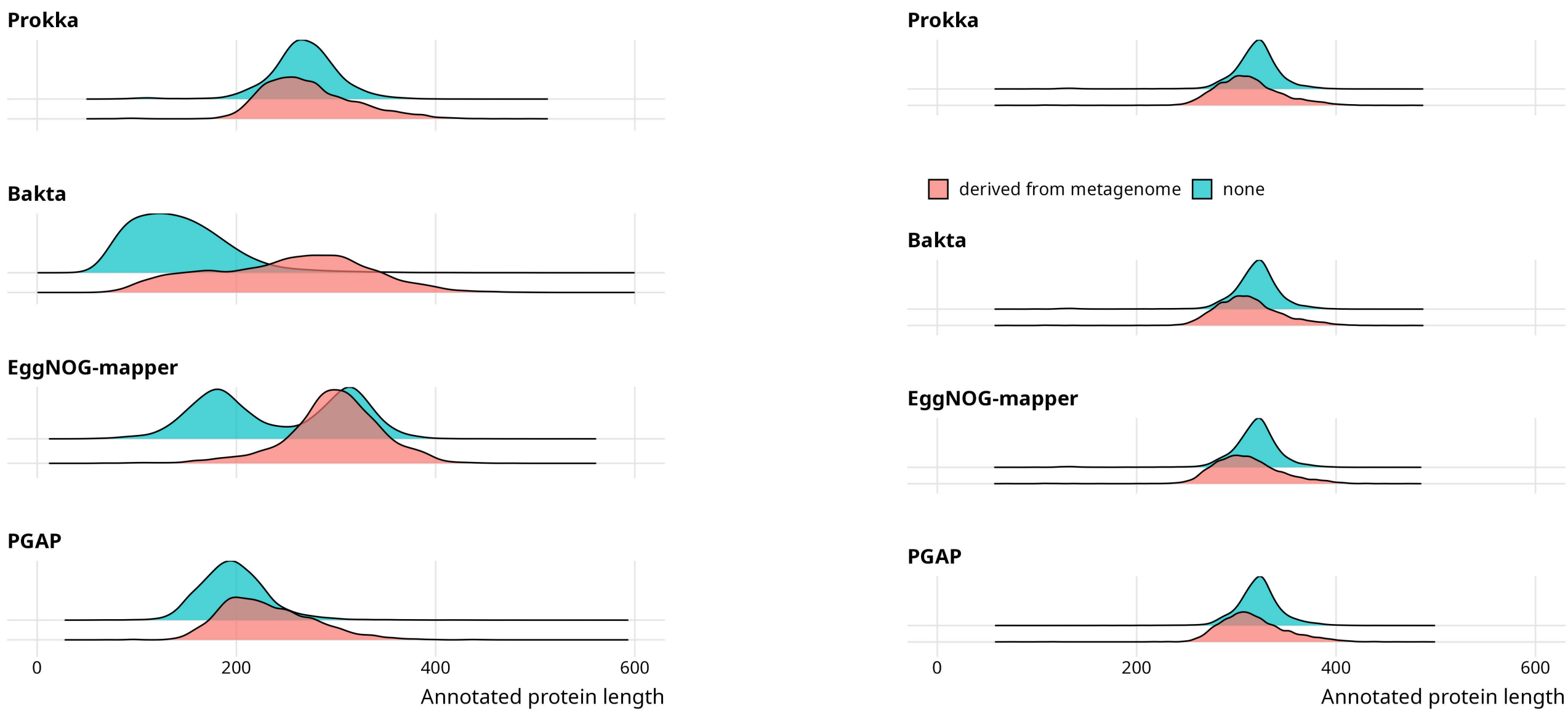

Fig. S18: Comparison of metagenomic protein lengths across the analyzed annotation tools in the Bacteria kingdom. Plot (a) shows the distribution of hypothetical protein lengths, while plot (b) includes all protein lengths.

a) Length of undescribed proteins in *E. coli*

b) Length of all proteins in *E. coli*

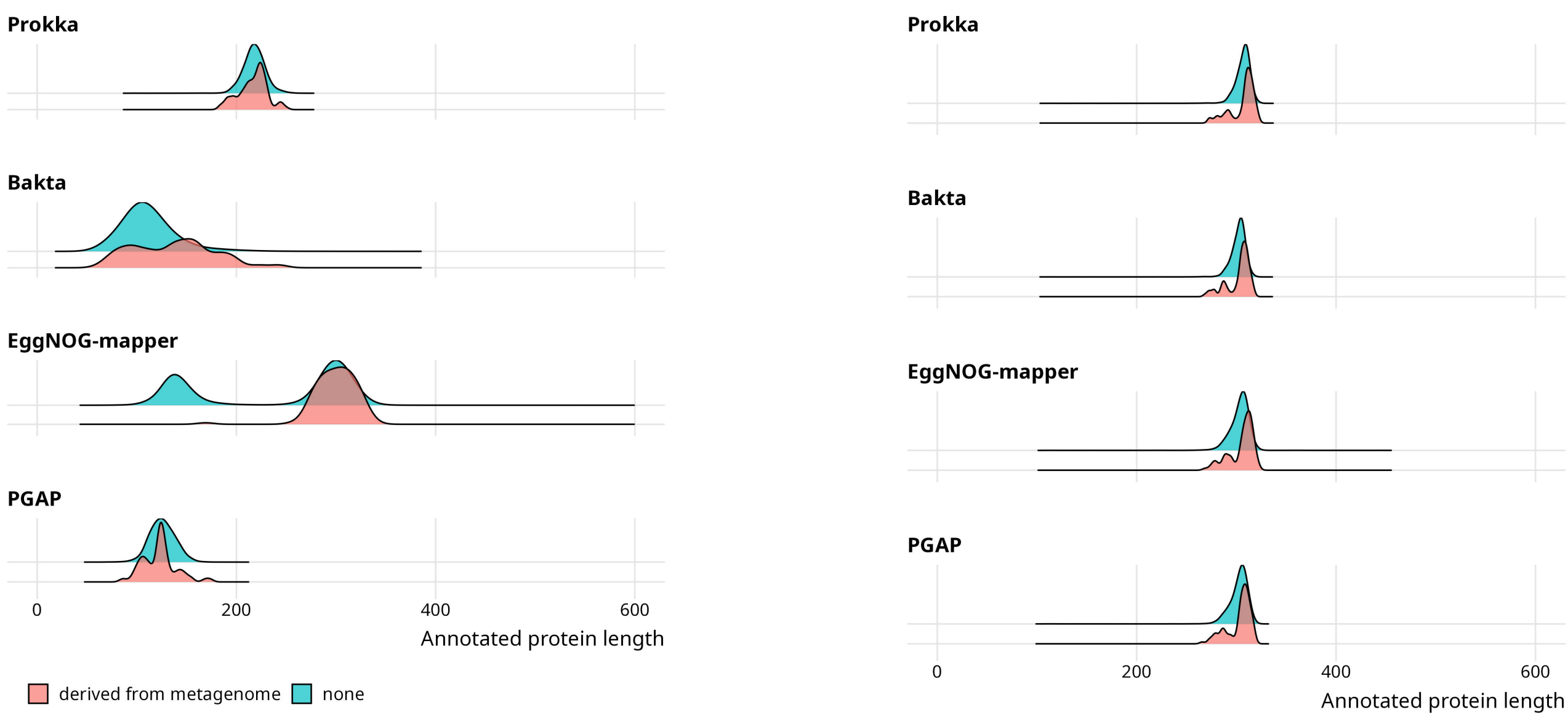

Fig. S19: Comparison of protein lengths across the analyzed annotation tools in *E. coli*. Plot (a) shows the distribution of undescribed protein lengths, while plot (b) includes all protein lengths.

7 Frameshifted genomes

7.1 Genome region comparison

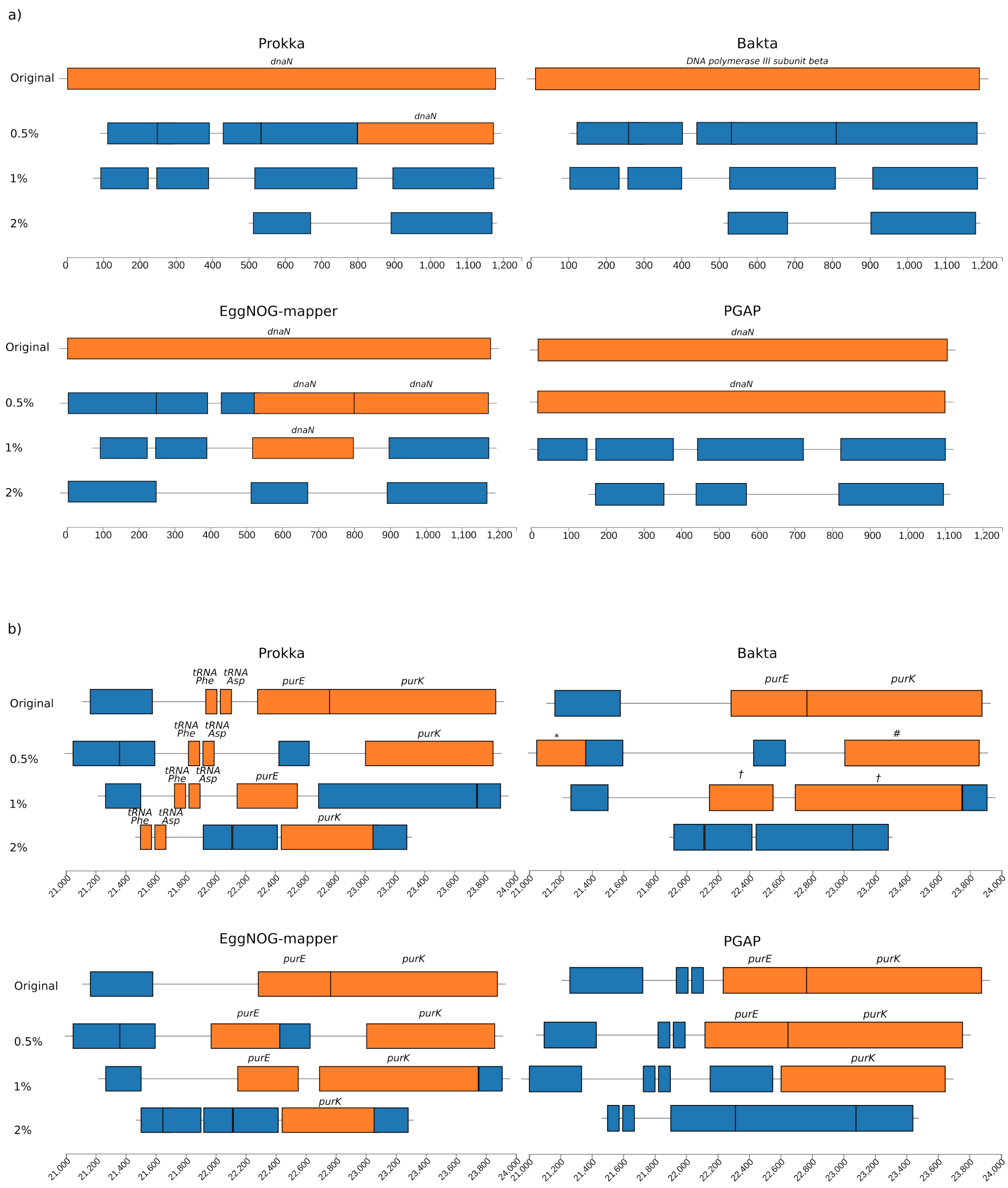

Fig. S20: Investigation of gene fragmentation between the original genome sequence (GCA_000008565.1) and sequences with increasing deletion rates (0.5%, 1%, and 2% of the full genome). Only gene features fully contained within the investigated regions are displayed. As deletion rates increase, the expected location of features shifts to the left in the plot. Blue features represent undescribed genes, while orange features represent described genes. **a)** Region 1–1,200 bp. **b)** Region 21,000–24,000 bp. Symbols for features with longer names are: * UDG domain-containing gene, # ATP-grasp domain-containing protein, + N5-carboxyaminoimidazole ribonucleotide synthase.

8 Temporal analysis

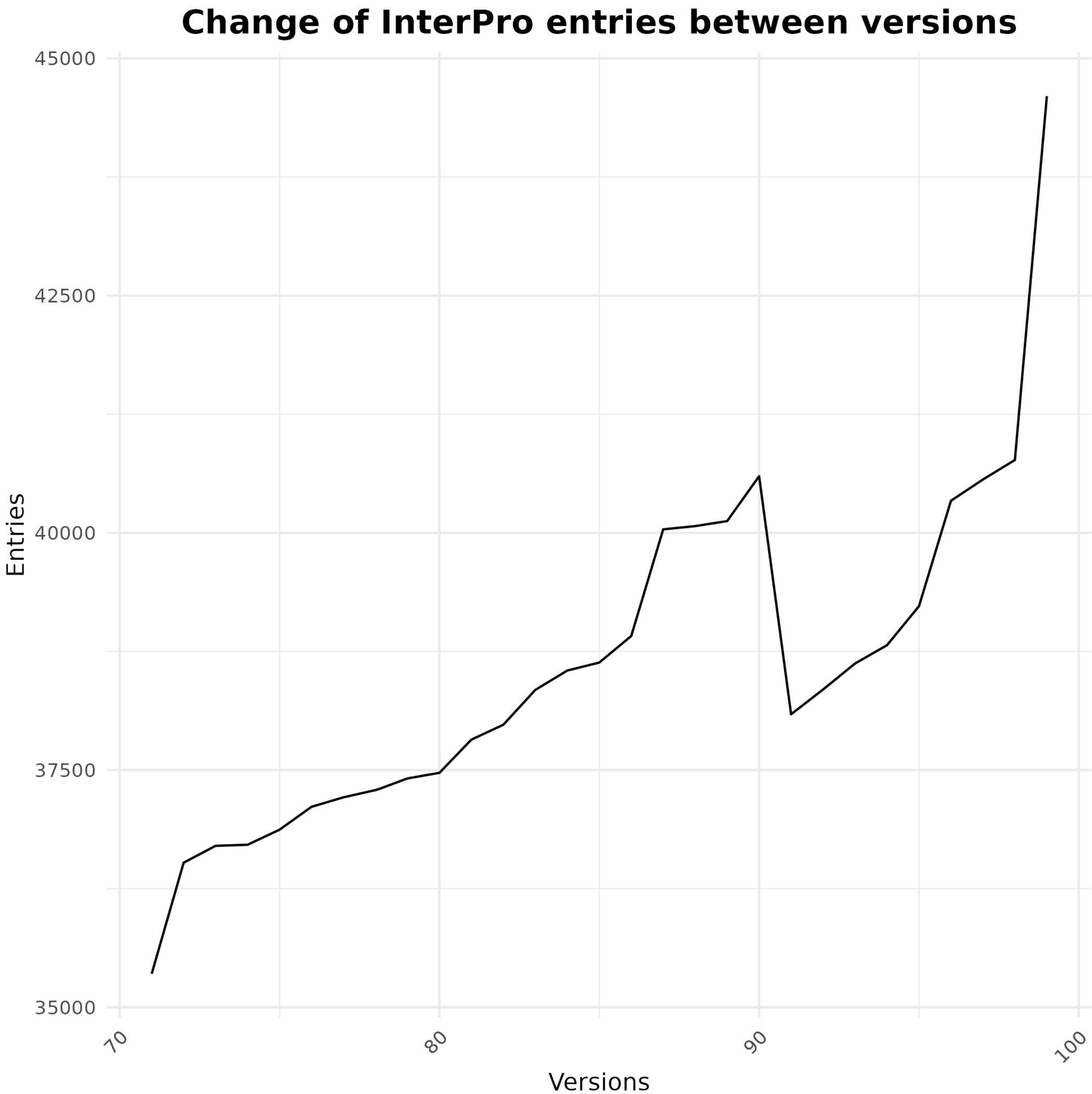

Fig. S21: Stability of protein counts in annotation results reported by NCBI across submission years. For example, InterPro entries increased substantially, from 38,088 to 46,035 over just two years (from InterPro 91.0 in October 2022 to 102.0 in October 2024).

8.1 Annotation performance over time

(a) Stability of protein counts in *E. coli* samples.

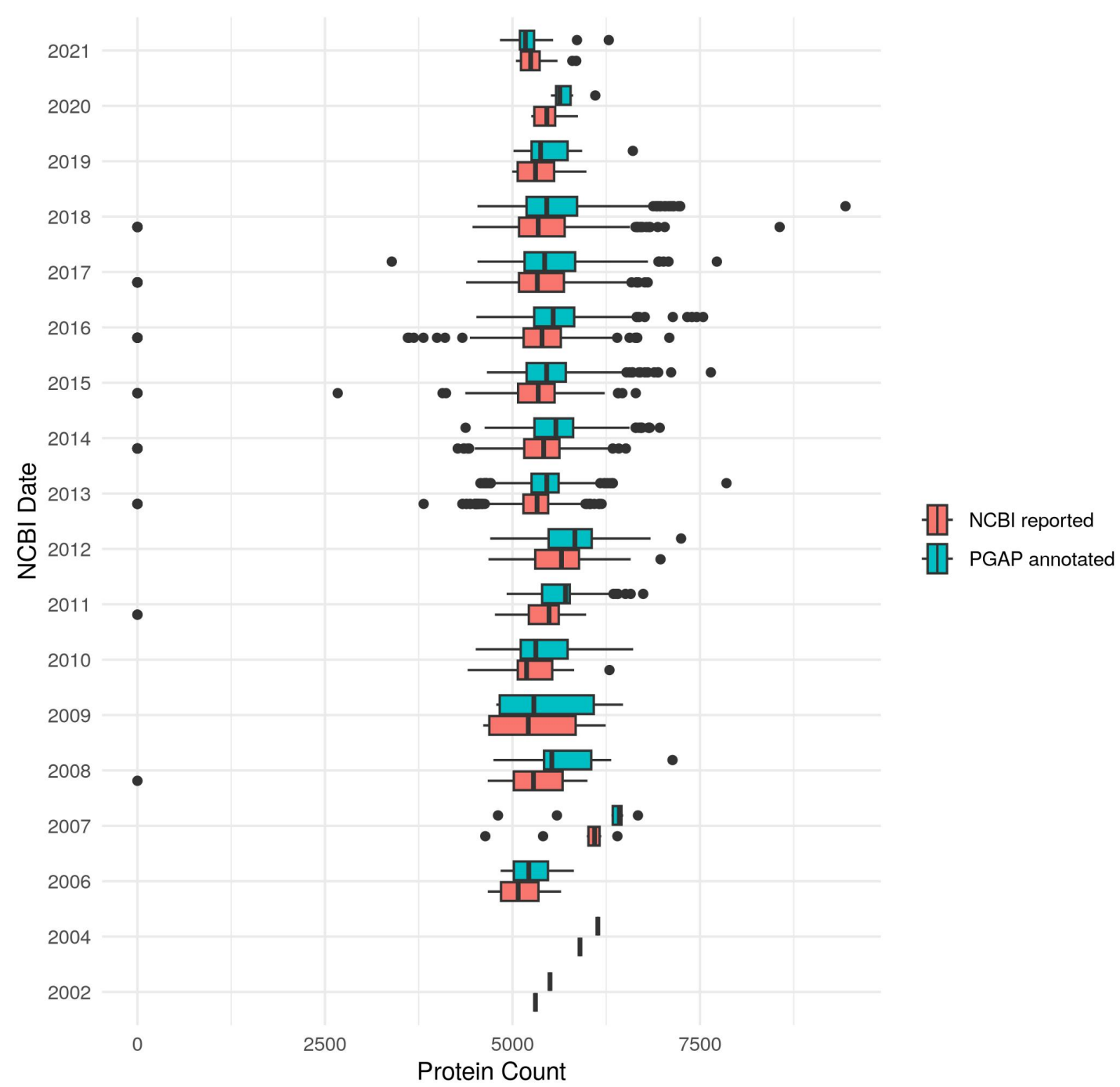

(b) Stability of protein counts in the Bacteria kingdom.

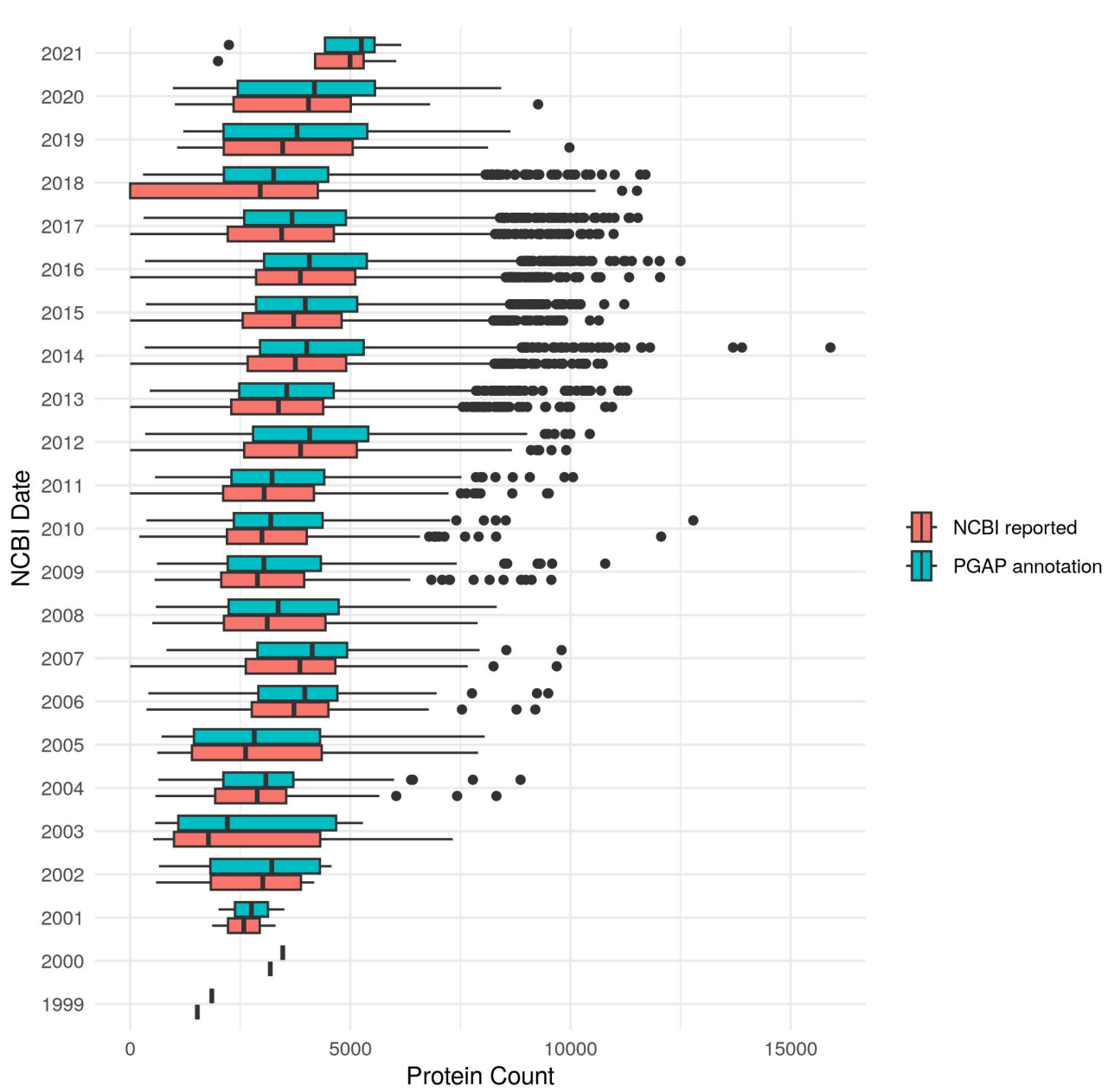

(c) Stability of protein counts in the Archaea kingdom.

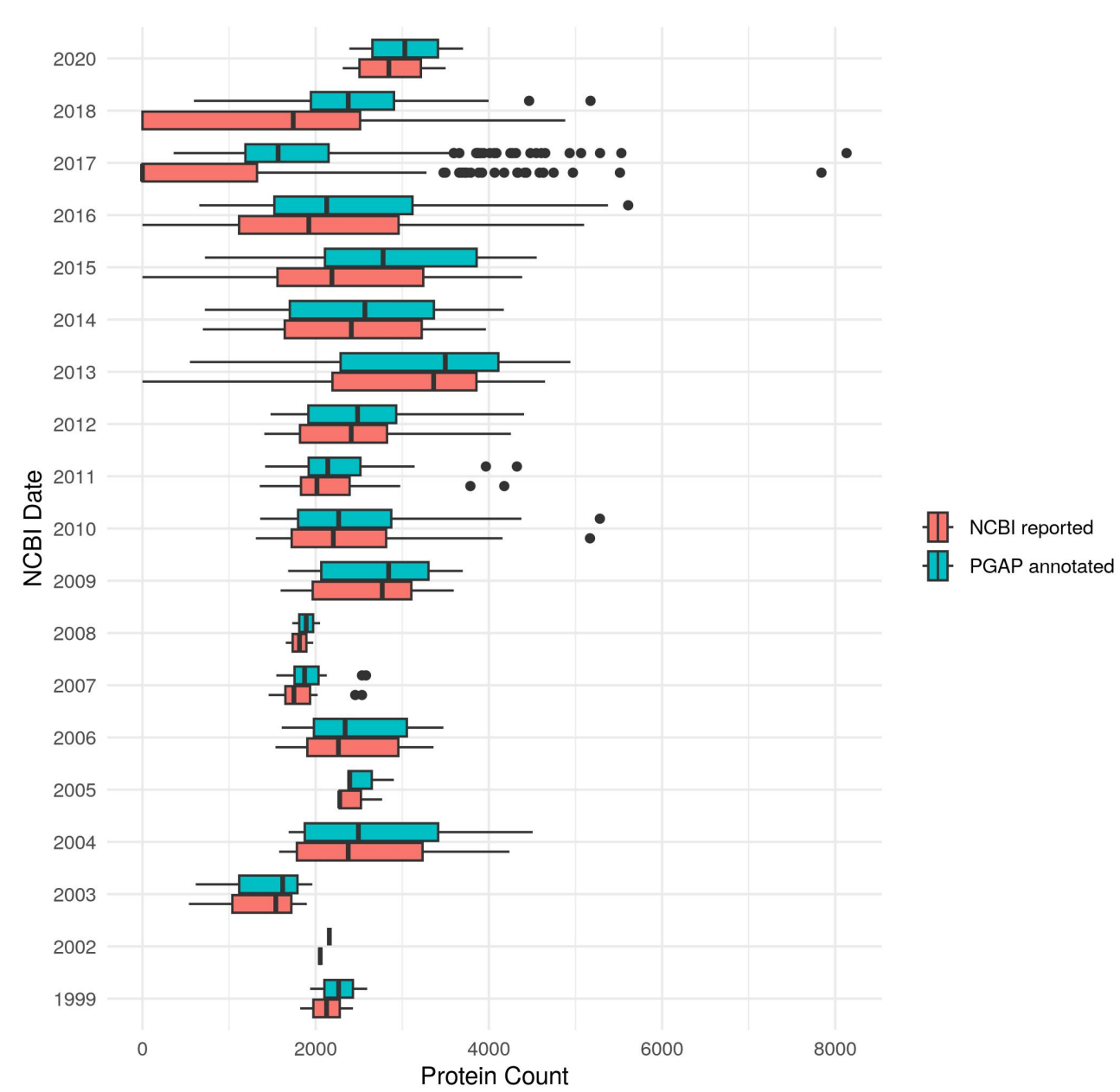

Fig. S22: Stability of reported protein counts across submission years for different taxonomic groups. (a) The *E. coli* group. (b) The Bacteria kingdom. (c) The Archaea kingdom.

8.2 Increase in unique species in the database over time

a) Increase in the number of unique bacterial species in GTDB per release.

b) Increase in the number of unique archaeal species in GTDB per release.

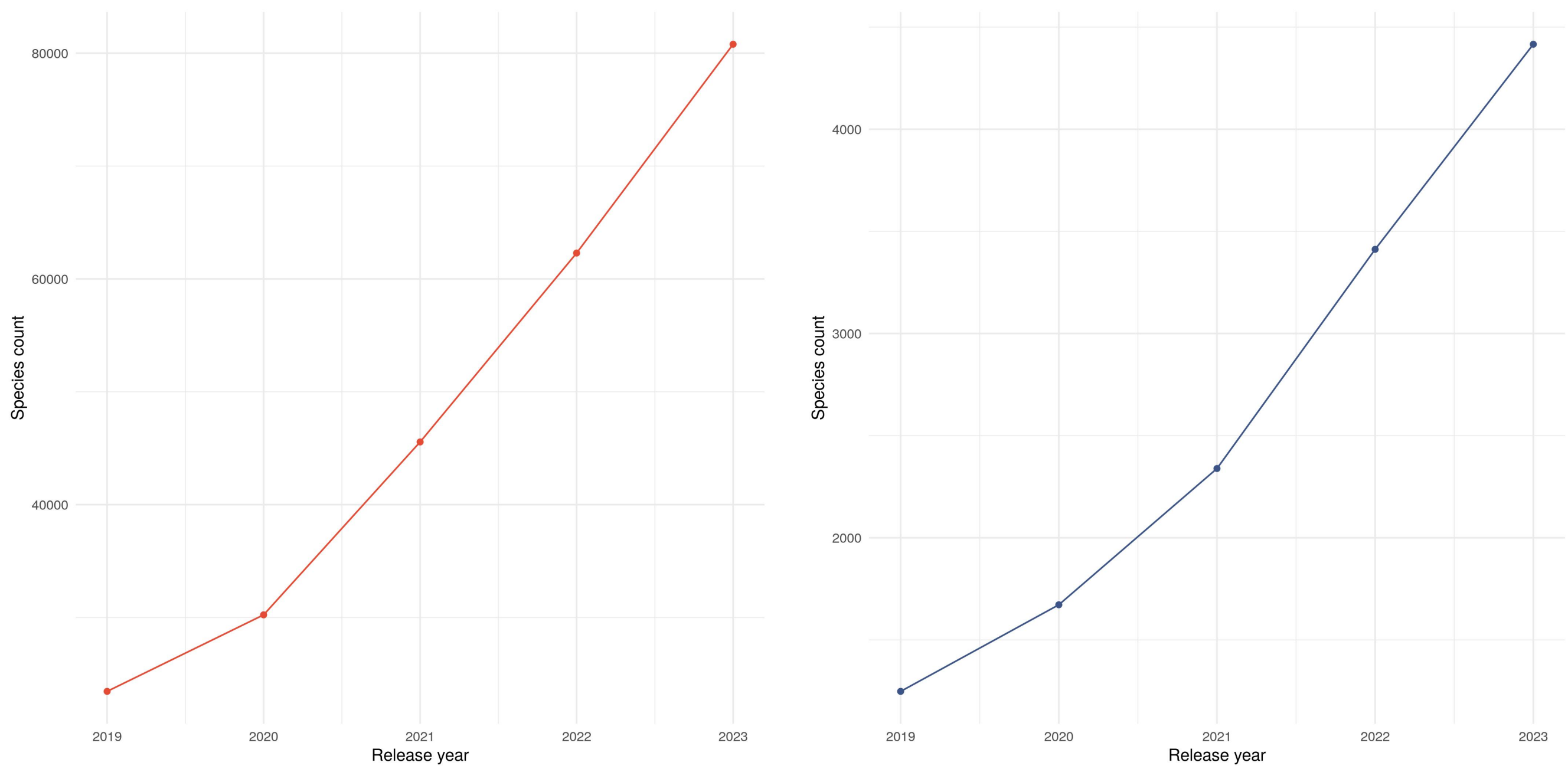

Fig. S23: Increase in the number of unique species per GTDB release for Bacteria and Archaea.

9 Workflow visualization

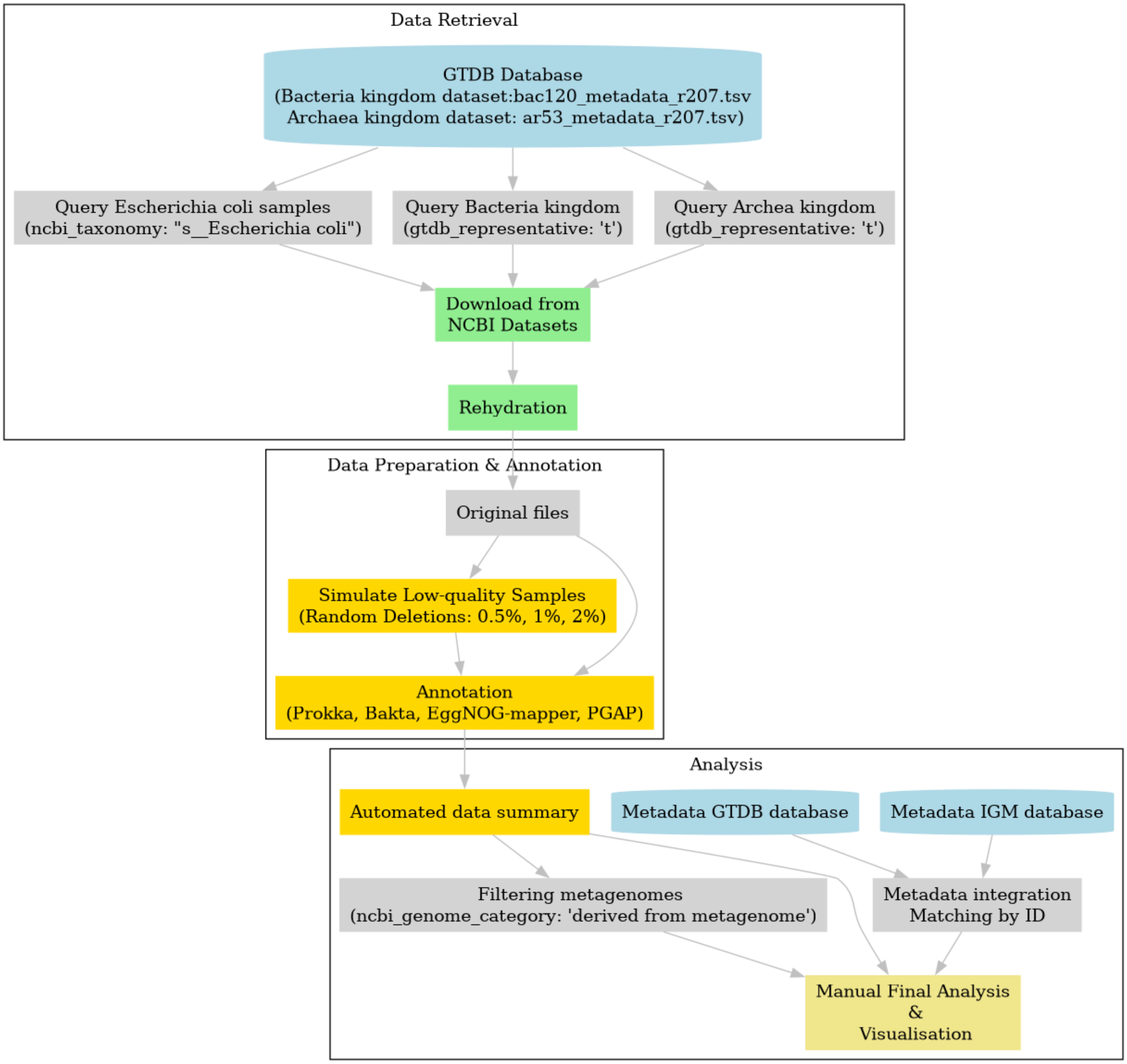

Fig. S24: Workflow diagram illustrating the analysis workflow. The diagram depicts genome querying and retrieval from the Genome Taxonomy Database (GTDB) and NCBI Datasets, followed by data preparation, annotation using Prokka, Bakta, EggNOG-mapper, and PGAP, automated data summarization using an R script, metadata integration, and manual final analysis and visualization. Gray arrows indicate the flow of data and processing steps in the workflow. Color-coded elements highlight different stages: light blue represents external databases, light green denotes use of the NCBI Datasets tool, gold indicates steps included in the Nextflow workflow, and khaki signifies the final manual analysis.
